# Atrial fibrillation: primary prevention, secondary prevention, and prevention of thromboembolic complications: part 1

**DOI:** 10.3389/fcvm.2023.1060030

**Published:** 2023-06-15

**Authors:** Richard G. Trohman, Henry D. Huang, Parikshit S. Sharma

**Affiliations:** Section of Electrophysiology, Division of Cardiology, Department of Internal Medicine, Rush University Medical Center, Chicago, IL, United States

**Keywords:** pathophysiology, epidemiology, lifestyle modification, pharmacological interventions, catheter ablation—atrial fibrillation

## Abstract

Atrial fibrillation (AF), is the most common sustained cardiac arrhythmia. It was once thought to be benign as long as the ventricular rate was controlled, however, AF is associated with significant cardiac morbidity and mortality. Increasing life expectancy driven by improved health care and decreased fertility rates has, in most of the world, resulted in the population aged ≥65 years growing more rapidly than the overall population. As the population ages, projections suggest that the burden of AF may increase more than 60% by 2050. Although considerable progress has been made in the treatment and management of AF, primary prevention, secondary prevention, and prevention of thromboembolic complications remain a work in progress. This narrative review was facilitated by a MEDLINE search to identify peer-reviewed clinical trials, randomized controlled trials, meta-analyses, and other clinically relevant studies. The search was limited to English-language reports published between 1950 and 2021. *Atrial fibrillation was searched via the terms primary prevention, hyperthyroidism, Wolff-Parkinson-White syndrome, catheter ablation, surgical ablation, hybrid ablation, stroke prevention, anticoagulation, left atrial occlusion and atrial excision.* Google and Google scholar as well as bibliographies of identified articles were reviewed for additional references. In these two manuscripts, we discuss the current strategies available to prevent AF, then compare noninvasive and invasive treatment strategies to diminish AF recurrence. In addition, we examine the pharmacological, percutaneous device and surgical approaches to prevent stroke as well as other types of thromboembolic events.

## Introduction

Atrial fibrillation (AF) is the most common sustained cardiac arrhythmia and is associated with significant morbidity and mortality (1). A 2010 estimate suggested that as the global population ages, AF is predicted to affect 6–12 million individuals in the United States by 2050, 17.9 million Europeans by 2060, and may exceed 70 million in Asia alone by 2050 (2–6).

In addition to the aging global population AF's increasing prevalence has been driven by a high burden of risk factors and comorbidities, thereby raising significant issues about the use of healthcare systems and economic costs (7). This review will focus on modifiable risk factors for AF, prevention of AF recurrence (including antiarrhythmic drug therapy, catheter, surgical, and hybrid ablation), and stroke prevention.

## Pathophysiology

AF is a complex disorder with shared environmental and genetic factors contributing to its pathogenesis. Three broad genetic approaches, that are not mutually exclusive, have been applied to AF. Linkage analysis uses families with Mendelian forms of AF. Genome-wide association studies (GWAS) use genotyping array data. Analyses of coding variation are gathered from whole-exome or whole-genome sequencing data (8).

Rapid progress has identified many common variant loci in GWAS for AF, yet major challenges remain in moving from disease associations to specific mechanisms. Recent genome and exome-based sequencing studies have identified *TTN* as the most common gene associated with mutations in individuals with AF. Future studies will aim to explore application of polygenic risk scores (PRS) to clinical care, building out genetic studies in non-Europeans, and further expand single-cell sequencing and genomic technologies in tissues and cells related to AF. Refinement of AF's genetic basis ultimately will facilitate identification of new therapeutic targets and enable more precise risk stratification for this tachyarrhythmia (8).

Four main pathophysiological mechanisms contribute to AF: electrical remodeling, structural remodeling, autonomic nervous system changes, and Ca^++^ handling abnormalities. These may result from cardiac disease fostering AF development. In turn, AF-induced atrial remodeling enhances cardiac vulnerability to AF induction and maintenance (9) (Table 1).

**Table 1:** Pathophysiologic mechanisms.

Electrical remodeling	Structural remodeling	Autonomic nervous system changes	Ca^++^ handling abnormalities
Down-regulation of I_CaL_	Atrial enlargement-key determinant of the persistent AF-maintaining reentry	Adrenergic activation increases risk of delayed afterdepolarizations (DADs) and formation of ectopic activity	Ca^++^ signaling plays a role in electrical remodeling
Up-regulation I_K1_	Atrial fibrosis → local conduction disturbances, promotes AF	Autonomic hyperinnervation is a consequence of AF-related remodeling	CaMKII activation promotes DADs/triggered activity CaMKII phosphorylation also activates downstream effectors of remodeling
I_KACh_, mediates acetylcholine effects and underlies the ability of vagal activation to promote AF	AF promotes atrial fibrosis which contributes to therapeutic resistance in longstanding AF		Protein kinase C is stimulated by an increased intracellular Ca^++^ and induces downstream signaling cascades controlling diverse functions such as fibroblast activation, cardiomyocyte hypertrophy, and inflammation
AF suppresses I_KACh_ but enhances a constitutive form (I_KAChc_), promoting AF maintenance			Ca^++^ signaling plays a role in structural remodeling; evidence points to a key role of cell Ca^++^ in atrial structural remodeling
Up-regulation of the small conductance Ca^++^-activated K^+^ channel			CaMKII hyperphosphorylation of RyR2, results in Ca^++^ leak from the sarcoplasmic reticulum; blockade prevents structural remodeling and AF in animal models
Altered gap junction function contributes to AF-induced remodeling^a^			

^a^ Mutations in *GJA5* underlie cases of idiopathic AF. GJA5 promoter sequence variants are associated with AF vulnerability.

Adapted from reference (9) with permission.

There is extensive evidence that initiation and maintenance of AF involves atrial ectopic triggers and a substrate prone to reentry. The pulmonary veins (PVs) play a central role as both ectopic sources and zones of reentry. Disturbances in conduction related to tissue fibrosis and/or connexin-abnormalities predispose to reentry. Abbreviated refractoriness is a potential contributor (1). Autonomic tone is a key regulator (1, 10). However, this entity is a diagnosis of exclusion and may be related to the effort made to identify underlying comorbidities. In patients with structural heart disease, paroxysms more typically occur during sympathetic predominance (8).

Two animal models demonstrated that sustained AF was inducible after rapid atrial pacing in dogs (11, 12). In the later study the right atrial refractory period decreased and an increase in atrial area of at least 40% was necessary to induce sustained AF (12). The phrase “atrial fibrillation begets atrial fibrillation” was introduced in a goat model in 1995. Initial induction of AF produced episodes lasting seconds. However, artificial maintenance of AF *via* rapid pacing led to marked shortening of the atrial effective refractory period (AERP) and development of sustained AF episodes. The authors noted that the presence of multiple wavelets needed to sustain AF would correlate with atrial enlargement because the number of circuits in the atria increases with the square of the atrial diameter (13).

A study of 35 patients with accessory pathways and no history of AF evaluated the effect of induction of brief AF episodes within 30 min post successful ablation. Under baseline conditions, the PVs demonstrated significantly longer ERPs compared to the atria (PVs vs. LA: 248 ± 27 ms vs. 233 ± 23 ms; *P* = 0.021) and right atrium 248 ± 27 ms vs. 207 ± 24 ms; *P* < 0.001. All 4 PVs demonstrated a significantly shorter ERP after the AF exposure compared with their baseline ERP. The mean ERP of all PVs decreased to a significantly greater extent as compared to the atria (PVs vs. RA: 37 ± 34 ms vs. 17 ± 19 ms; *P* = 0.005 and PVs vs. LA: 37 ± 34 ms vs. 19 ± 20 ms; *P* = 0.009). The authors suggested that because these changes are significantly more pronounced in the PVs as compared with the atria, they provide evidence that “AF begets AF in the PVs” (14).

Risk factors and possible causes of AF include advanced age, male sex, left ventricular dysfunction/heart failure (HF), hypertrophic cardiomyopathy/left ventricular hypertrophy, ischemic heart disease, rheumatic/valvular heart disease, hypertension, diabetes, left atrial dilatation, and smoking/pulmonary disease. Obesity (obese individuals have a 51% greater risk of AF development compared with non-obese counterparts) (15), sleep apnea, congenital heart disease, diuretic use, cardiothoracic surgery, hyperthyroidism, pericarditis, binge drinking/alcohol poisoning, autonomic dysfunction, sinus node dysfunction, and supraventricular tachyarrhythmias (especially those mediated *via* accessory pathways when manifest preexcitation is present) (16) have also been associated with AF (17).

Hypertensive heart disease (67%–76%) and coronary (ischemic) heart disease are the most common underlying disorders in AF patients from developed nations. Rheumatic heart disease, now uncommon in developed nations, is associated with a much higher incidence of AF (18).

This review will focus on modifiable risk factors for AF, prevention of AF incidence and recurrence (including antiarrhythmic drug therapy, as well as catheter, surgical and hybrid ablation), prevention of AF after cardiothoracic surgery, plus special circumstances where treatment/elimination of the underlying condition may reduce or eliminate AF burden and stroke prevention in AF.

## Epidemiology of atrial fibrillation

AF incidence doubles with each decade of adult life (19); increasing from 2 to 3 new cases per 1,000 persons/year at ages <64 years, to ∼19.2 per 1,000 person-years in those 65–74 years old and reaching as high as 31.4–38.0 in octogenarians (19–21). Clinical and community-based studies report a 20%–50% lower age- and sex-adjusted risk of clinically detected atrial fibrillation or flutter in African Americans than in whites. Racial disparities in the treatment of AF patients may account for this difference (22). However, African Americans have a higher prevalence of AF risk factors including hypertension, obesity, and diabetes, and a higher stroke risk (22). Nevertheless, in an inception cohort of 3,507 new patients with AF, blacks with new-onset AF were more likely to have an ischemic stroke before or after the diagnosis of AF (23).

In an attempt to clarify these apparent discrepancies, 1,556 patients from the Multi-Ethnic Study of Atherosclerosis (MESA) participated in an ancillary study (22). Among 1,556 participants, 41% were white, 25% African American, 21% Hispanic, 14% Chinese and 61% were women. After 14.4 years of follow-up, the prevalence of clinically-detected AF was 11.3% in whites, 6.6% in African Americans, 7.8% in Hispanics, and 9.9% in the Chinese. The difference in clinically detected AF between whites and African Americans was statistically significant (*P* < 0.001). The prevalence of clinically-detected AF did not differ significantly between Hispanics or Chinese and white patients. Fourteen days of ambulatory ECG monitoring was then performed. The investigators reported episodes of monitor-detected AF lasting over 24 h. The proportions with monitor-detected AF were similar in the four racial/ethnic groups. In analyses limited to those without clinically-detected AF, the proportion with monitor-detected AF was again similar in the four race/ethnic groups. In patients with clinically-detected AF, the proportion with monitor-detected AF was higher in African American and Hispanic participants (42% and 40%) than in white and Chinese participants (28% and 19%). These results supported the hypothesis of differential detection by race/ethnicity in the clinical recognition of AF (22). In contrast, Osman and colleagues calculated the prevalence of AF among white, Hispanic, and Black patients in 6 common hospital admission categories. In order to adjust for differences in risk profile, they used a multilevel mixed-effects logistic regression model that included age, sex, hypertension, hyperlipidemia, diabetes, vascular disease, carotid stenosis, coronary disease, prior sternotomy, and smoking. More than 5 million weighted hospitalizations were included. AF prevalence among Black and Hispanic patients was significantly lower than in white patients across all admission categories. The authors acknowledged that a large difference in hospitalization rates across different races could neutralize the differences in AF prevalence in this study. However, they pointed out that this was highly unlikely for two reasons: (1) prior studies using granular rhythm detection methods in ambulatory patients showed similarly lower AF rates among non-white patients, and (2) the incidence of hospitalizations for certain diseases (included in the analysis) was higher in non-white patients. For example, stroke and pulmonary embolism were more common in black vs. white patients, which made "undersampling" of these patients due to the inclusion of inpatients admissions only unlikely (24). In the Atherosclerosis Risk in Communities (ARIC) Study 15,343 participants (aged 45–64) without baseline AF were recruited from 1987 to 1989 and followed until 2014. In this large cohort, the lifetime risk of AF was ∼1 in 3 among whites and 1 in 5 among African Americans, however risk was inversely proportional to socioeconomic status (25).

In Europe, studies performed in the global population between 2007 and 2013 reported an incidence of AF ranging from 0.23 per 1,000 person/years in Iceland to 0.41 in Germany and 0.9 in Scotland (26). A 2019 study from Italy reported an AF prevalence of 7.3% in individuals over the age of 65 and predicted an increase in the European Union by 89% in 2060 (27). AF affects an estimated 11 million people in Europe and by 2050, Europe is projected to have the greatest increase in AF (to 18 million people) compared to other regions globally (28). In Asia, the incidence and prevalence of AF has also increased in recent years, although great variability still exists among Asian nations. A systematic review of AF in Asian countries (mainly from China, Japan, and Korea) found an incidence of 5.38 per 1,000 person-years after meta-analyzing 10 studies from three countries (29).

Caution should be exercised in evaluation of AF in Europe because the continent is not a homogeneous entity. A temporal (1990–2017) analysis of data from the 2017 Global Burden of Disease Database was performed to evaluate changing trends in AF incidence and mortality in 20 European countries. The analysis identified no across-the-board trend descriptive of all nations. Mortality-to-incidence ratios were calculated for each country. Although AF incidence was higher in men, mortality to incidence ratios were higher in all countries in women (30).

These differences were attributed either to biological differences or health care inequality. Surprisingly, a national low gross domestic product (predominantly in Eastern European and the Baltic nations) was related to lower AF incidence and AF-related mortality. The authors speculated that residents in high gross domestic product countries receive better healthcare and live long enough to be diagnosed with and/or suffer from the serious complications of AF (a survivor effect). In addition, the authors speculated that low gross domestic product countries had poorer health systems resulting in underdiagnosis/underreporting of AF and its consequences (30). Thus, it was also possible that improved awareness and detection of AF may have contributed to higher AF associated mortality, in the higher GDP countries (31).

The overall prevalence of AF is higher in men than women. However, because women typically live longer than men, there are nearly twice as many women as men aged >75 years. Beyond the age of 75 years (the median onset age for AF), ∼60% of individuals with AF are women (32, 33). Men with AF have more ischemic heart disease and women have more valvular disease (34). The Canadian Registry of Atrial Fibrillation (CARAF) database showed that women with AF had an increased prevalence of hypertension and thyroid dysfunction (35, 36). In the absence of anticoagulation, women are at higher risk than men for AF-related thromboembolism (37). A large meta-analysis clearly established that women with AF carry a persistently higher stroke risk, even when adequate anticoagulation is prescribed (38). Sex-associated risk, during treatment with direct oral anticoagulants (DOACs) is not completely understood (39).

## Modern approach to atrial fibrillation management and prevention

**Figure 1:**
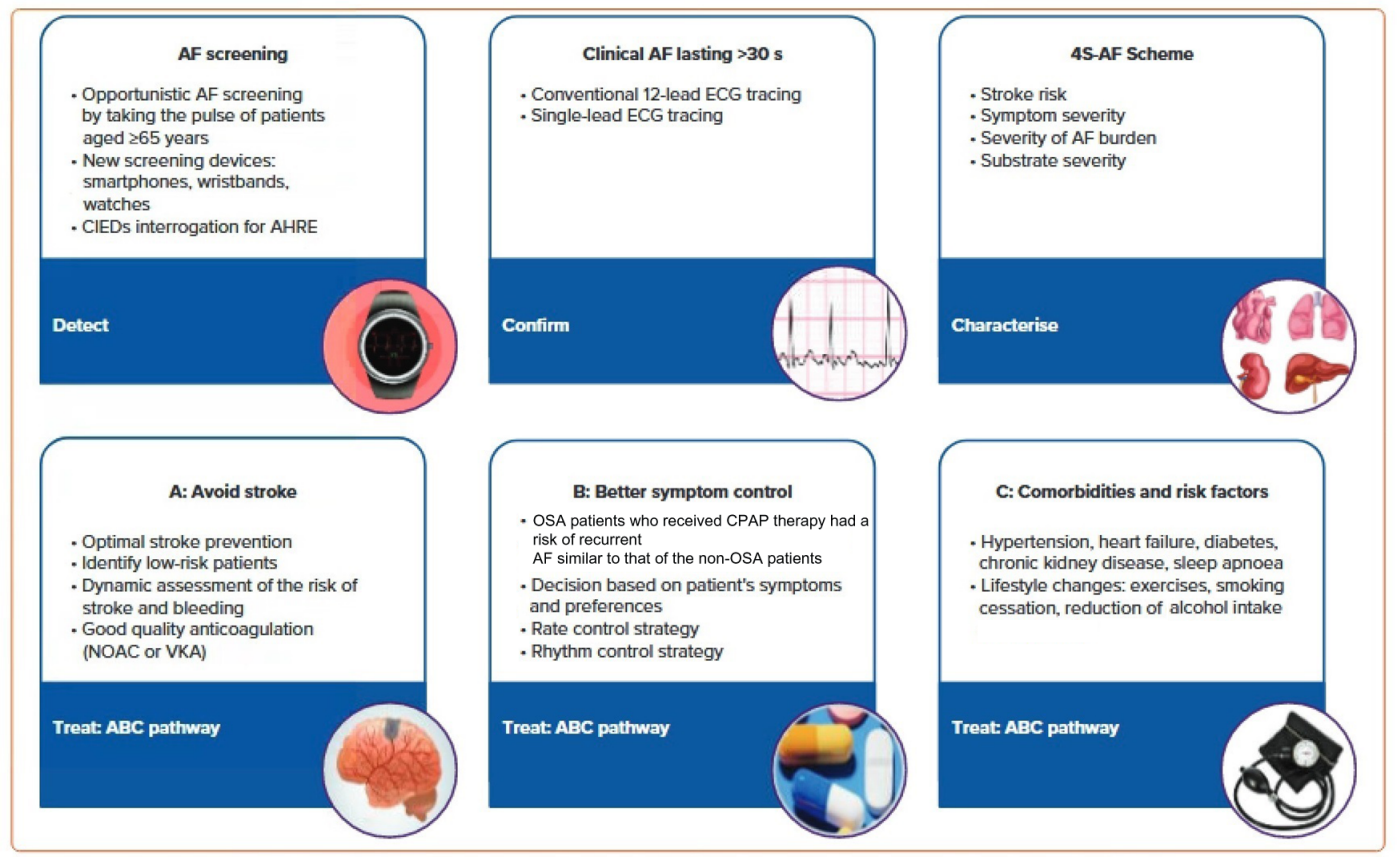
AF patient management based on the 2020 ESC guidelines. AHRE, atrial high-rate episode; CIEDs, cardiac implantable electronic devices; EDG, electrocardiogram; NOAC, non-vitamin K antagonist; VKA, vitamin K antagonist. Reproduced from reference (7) with permission.

The 2020 ESC Guidelines on the Diagnosis and Management of AF have provided a systematic approach to AF detection, characterization, and management (see Figure 1) (7, 40). Considerable progress has been made in AF detection (including asymptomatic AF episodes). A variety of wearable technologies and several implantable loop recorders are now available to detect and record AF episodes (7).

Characterization was initially proposed as a paradigm shift to address specific domains having treatment and prognostic implications. The 4S-AF scheme (stroke risk, symptom severity, severity of AF burden and substrate for AF), a novel pathophysiology-based characterization of AF patients, can be employed in daily practice and supports decision-making regarding prescription of oral anticoagulation (OAC), rate or rhythm control strategies (AF ablation or antiarrhythmic drugs) as well as management of concomitant risk factors and comorbidities (7).

The AF Better Care (ABC) pathway (A: avoid stroke; B: better symptom control; C: comorbidities and risk factors) (7, 41) streamlines management of AF patients (see Figure 1 for additional details). Treatment strategies that are consistent with the ABC pathway have improved outcomes for AF patients by reducing the rates of rehospitalization (see below), cardiovascular events and all-cause mortality (7, 42, 43).

**Figure 2:**
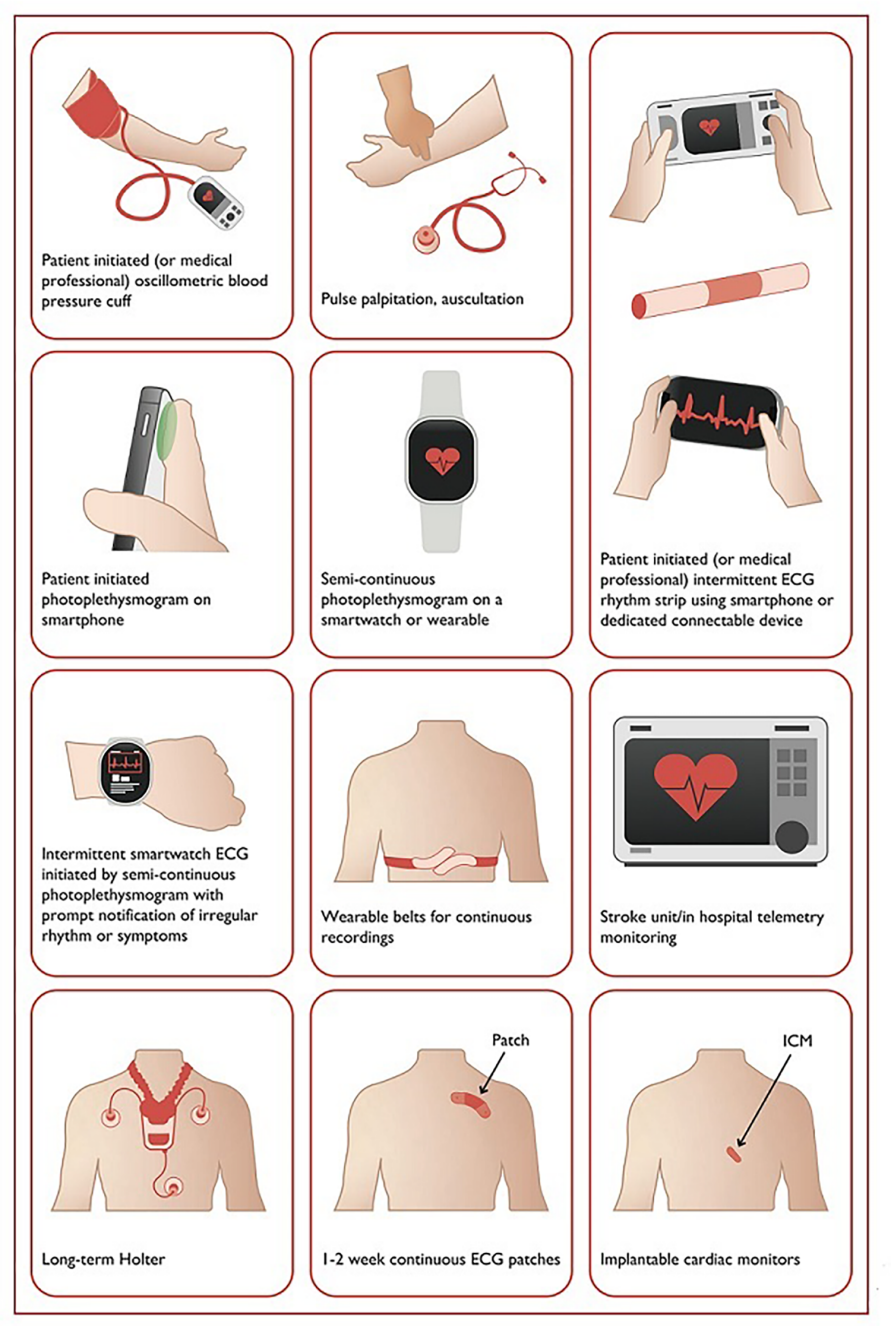
Systems used for AF screening. Reproduced from reference (40) with permission.

The mAF-App II Trial Investigators randomized Chinese patients from 40 cities to investigate the merits of this more holistic and integrated approach to AF management. Patients (1676) received integrated care based on a mobile AF Application (mAFA) incorporating the ABC (Atrial Fibrillation Better Care) Pathway or usual care (1678). The composite outcome of ischemic stroke/systemic thromboembolism, death, and rehospitalization was lower with the mAFA intervention compared with usual care (*P* < 0.001). Rates of rehospitalization were also lower with the mAFA intervention (*P* < 0.001). Subgroup analyses by sex, age, AF type, risk score, and comorbidities demonstrated consistently lower hazard ratios for the composite outcome for patients receiving the mAFA intervention compared with usual care (all *P*-values <0.05) (43). Figure 2 illustrates various AF detection modalities (40).

In addition, the benefits of the 4S-AF scheme and ABC pathway adherent care have been clearly demonstrated to be associated with reduction in cardiovascular death, major bleeding, and stroke compared to usual care. The ABC pathway is also recommended in the 2021 Asia-Pacific Heart Rhythm Society Guidelines (7, 44).

## Are there readily reversible AF precipitants?

Past consensus guidelines stated “AF may be related to acute, temporary causes, including alcohol intake (‘holiday heart syndrome’), surgery, electrocution, MI, pericarditis, myocarditis, pulmonary embolism or other pulmonary diseases, hyperthyroidism, and other metabolic disorders. In such cases, successful treatment of the underlying condition often eliminates AF” (45).

Additional secondary precipitants including fever/infection and acute alcohol consumption were identified among 1,409 Framingham Heart Study patients with new-onset AF. AF recurred in 544 of 846 eligible individuals without permanent AF (5-, 10-, and 15-year recurrences of 42%, 56%, and 62% with vs. 59%, 69%, and 71% without secondary precipitants). Although the 15-year incidence of AF recurrence was significantly lower among participants with secondary precipitants, AF eventually recurred in the majority with of individuals with “reversible causes” (46). Although iron deficiency and anemia are common conditions in AF patients, a clearcut cause and effect relationship is not well established (47).

It is commonly assumed that AF related to accessory pathways and hyperthyroidism is reversible. These assumptions are not entirely accurate.

Hyperthyroidism is a known precipitant of AF. However, <1% of AF cases are secondary to acute hyperthyroidism (48, 49). Pharmacologic rhythm control is not usually recommended. In one report, 8.3% of patients with new-onset hyperthyroidism developed AF or atrial flutter within 30 days (48, 50). In a retrospective study including 163 patients with hyperthyroidism and AF, 101 (62%) reverted to normal sinus rhythm after becoming euthyroid (51). Most (75%) who reverted to sinus rhythm did so within 3 weeks. Increased risk of hyperthyroidism induced AF is associated with male gender, advancing age, coronary artery disease, congestive heart failure and valvular heart disease, which are also traditional AF risk factors in the general population (48, 50, 52).

Whether the risk of developing stroke and thrombotic episodes is increased in hyperthyroidism induced AF is controversial. It has been suggested that thromboembolic events are primarily related to advanced age. Souza et al. noted that among patients younger than 65 years of age with atrial fibrillation related to hyperthyroidism, there was no association between clinical risk factors with transesophageal markers of a thrombogenic milieu. In this study it was found that only age was an accurate predictor of a thrombogenic milieu with other risk factors having a low yield (53). In the very large Swedish Atrial Fibrillation cohort study, hyperthyroidism was not an independent stroke risk factor (54). A 2015 study reported that in patients with hyperthyroidism related-AF and a CHA_2_DS_2_-VASc score of 0, the risk of ischemic stroke was virtually nil, irrespective of the type of AF (self-limiting or not), thus the use of anticoagulation therapy was deemed inappropriate. However, it also noted that among patients with CHA_2_DS_2_-VASc score ≥1, warfarin therapy was associated with a reduced ischemic stroke risk only in those with non–self-limiting AF, not those with self-limiting AF (55). A large more recent retrospective cohort study suggested that hyperthyroidism-related AF patients have a greater risk of ischemic stroke and systemic embolism like nonthyroidal AF, especially when initially diagnosed. The risk was reduced by treating hyperthyroidism (56). The 2020 ESC Guidelines on the Diagnosis and Management of AF recommends anticoagulation based on standard CHA_2_DS_2_-VASC score criteria (40).

The long-term prognosis of hyperthyroidism induced AF is not well known (due to limited long term follow up). Nevertheless, atrial premature beats are more frequent in thyrotoxic patients compared to matched controls before and after treatment. This raises the possibility that the risk of AF recurrence and the long-term prognosis are similar in euthyroid and hyperthyroid AF groups (48, 57).

AF is a potentially life-threatening arrhythmia in patients with rapid antegrade conduction (pre-excitation) *via* an accessory pathway (AP) since it may lead to ventricular fibrillation (VF). Patients with APs that only conduct in the retrograde direction may also develop AF (58). However, in the absence of preexcitation, degeneration of AF to VF is extremely rare (59).

Proposed mechanisms of AF initiation in patients with APs include enhanced atrial vulnerability and degeneration of atrioventricular reentrant tachycardia into atrial fibrillation. Although surgical studies have suggested virtually no recurrence of AF after AP resection, radiofrequency catheter ablation has become the treatment of choice for symptomatic patients (57).

Dagres and colleagues compared AF recurrence rates of in 91 patients with history of paroxysmal AF who underwent successful AP catheter ablation to a control group consisting of 100 consecutive patients without a history of paroxysmal AF who underwent successful radiofrequency ablation of an accessory pathway. During a mean follow-up of 23.9 ± 12.3 months, AF recurrence/occurrence was significantly lower in the control group than in the study group (4 of 100 patients in the control group patients vs. 18 of 91 study group patients, (*P* = 0.001). The authors only identified age >50 years as a significant independent predictor of atrial fibrillation recurrence (*P* = 0.02) (57).

## Primary prevention of atrial fibrillation

Primary prevention strategies for AF have not been well-explored. However, individuals with optimal cardiovascular health have a 62% lower risk of AF (59, 60). Many risk factors and underlying conditions predisposing to AF are also risk factors for other cardiovascular issues such as coronary artery disease (CAD), vascular disease, and HF. Patients with multiple risk factors are the most susceptible to AF and therefore, the most appropriate target population for primary prevention strategies (60). There is emerging evidence suggesting that addressing modifiable risk factors may be effective for primary (and secondary) AF prevention (61). Targeting predispositions (Figure 1) as soon as possible (preferably before AF becomes manifest clinically) may potentially avert or reverse atrial remodeling, thus preventing AF or limiting its progression. Interventions aimed at risk factors should be tailored to individual patient needs (7, 60, 61). Unfortunately, the efficacy of intensive lifestyle intervention in reducing incident AF remains to be fully established (see below) (62).

## Pharmacologic and dietary approaches

### Beta blockers, amiodarone, ivabradine: emphasis on postoperative AF

The efficacy of beta-blocking agents for AF rate control is well established (62–64). Most of the evidence for AF in primary prevention relates to prophylaxis against postoperative AF after cardiac surgery (62). Amiodarone, which produces noncompetitive β-blockade (65), has also been shown to be effective in this setting (66). A trial randomly assigned 316 patients to receive a 48-hour infusion of metoprolol, 1 to 3 mg/h, according to heart rate, or amiodarone, 15 mg/kg of body weight daily, with a maximum daily dose of 1,000 mg, starting 15 to 21 h after cardiac surgery. AF occurrence was similar in the two groups (67). While acknowledging the limitations of their study, the authors endorsed adherence to guidelines and recommended use of β-blockers as first-line prophylaxis of postoperative AF (67). It has been suggested that combining ivabradine with a beta blocker is more effective in prevention of postoperative AF than either agent alone (68, 69). This approach has not been widely adopted.

### Statins

In short term trials, statin treatment seemed to reduce the odds of an episode of atrial fibrillation however, longer term (mostly larger) trials of statin vs. control treatment were not associated with significant AF reduction (62, 70). Like beta-blockers, pre-operative statin prophylaxis reduces AF post cardiac surgery. A meta-analysis of nearly 800 patients revealed that pre-operative statin prophylaxis resulted in a 43% reduction in post-operative AF (62, 71). The duration of preoperative statin prophylaxis resulted in increased postoperative AF risk reduction (3% per day) (71).

### Omega-3 fatty acids and vitamin D

Omega-3 fatty acids [n-3 polyunsaturated fatty acids (n3-PUFA), such as fish oil] exert anti-inflammatory effects similar to statins (62). In the Cardiovascular Health Study, higher levels of circulating n3-PUFA were associated with a lower risk of incident AF (62, 72). Prospective observational data from the same study suggested consumption of broiled or baked fish, common n3-PUFA sources, was linearly linked to lower AF incidence (62, 73). Larger epidemiologic studies did not show a beneficial effect of fish intake on atrial arrhythmias (62, 74, 75). Small studies suggested that pre-operative oral and intravenous n3-PUFA demonstrated efficacy in AF prevention post cardiac surgery (62, 76, 77). More recent randomized trials did not reproduce the same results (62, 78, 79). Hence, clinical utility of n3-PUFA supplementation for primary prevention of AF has not been established (62).

Vitamin D is a natural antioxidant. Although some reports have suggested that antioxidant vitamin supplementation might play a role in preventing AF, there had been no clear-cut clinical evidence supporting its efficacy in primary AF prevention (62), because (like omega-3 fatty acids) large-scale, long-term randomized trial data was unavailable. However, in 2021, the efficacy of marine omega-3 fatty acid and vitamin D supplementation on the incidence of AF was reported in a large randomized clinical trial. Over 25,000 participants were randomized and included in the analysis. Participants were randomized to receive: the (1) marine omega-3 fatty acids, eicosapentaenoic acid (EPA) and docosahexaenoic acid (DHA) + vitamin D3; (2) EPA-DHA and placebo; (3) vitamin D3 and placebo; or (4) 2 placebos. In this large-scale, primary prevention trial, marine omega-3 fatty acids and/or vitamin D_3_ did not significantly reduce or increase the primary end point of incident AF compared with placebo during a median treatment duration of 5.3 years. There was no evidence of an interaction between the agents employed. The authors concluded their findings did not support use of supplemental EPA-DHA and/or vitamin D_3_ for primary prevention of AF (80).

### Protein consumption

Preliminary data from a secondary analysis of postmenopausal women from the Women's Health Initiative Clinical Trial and Observational Study suggests that protein consumption of 58–74 grams/day was associated with a statistically significant reduction in the risk of AF across all levels of physical activity (81). Additional information on diet and weight loss is discussed under secondary prevention below.

### Renin-angiotensin-aldosterone system (RAAS) inhibitors

Retrospective analyses of large, randomized trials suggested a role for renin-angiotensin-aldosterone system inhibitors in primary AF prevention. In the Trandolapril Cardiac Evaluation (TRACE) study, trandolapril treatment was associated with a 47% lower incidence of new-onset AF in post-MI patients with systolic dysfunction (62, 82). A subanalysis of the Studies of Left Ventricle Dysfunction (SOLVD) revealed a 78% risk reduction in AF in heart failure patients receiving enalapril compared to placebo (62, 83). A post-hoc analysis of the Losartan Intervention for Endpoint Reduction in Hypertension (LIFE) study, revealed a 33% risk reduction in new-onset AF in hypertensive patients treated with losartan comparison to atenolol (10, 62). In the Val-HEFT (Valsartan Heart Failure Trial) a 37% lower AF incidence was seen in heart failure patients treated with valsartan (62, 84). In the CHARM trial (Candesartan in Heart Failure: an Assessment of Reduction in Mortality and Morbidity), an 18% reduction in AF occurrence was seen in symptomatic heart failure patients treated with candesartan (62, 85). The Ongoing Telmisartan Alone and in Combination with Ramipril Global Endpoint Trial (ONTARGET) demonstrated a trivial (statistically insignificant) trend towards lower incidence of new-onset AF in high-risk hypertensive patients receiving combination telmisartan and ramipril therapy compared to either telmisartan or ramipril alone (62, 86). More recent trials are less encouraging. In the Heart Outcomes Prevention Evaluation (HOPE), ramipril had no effect on AF incidence compared to placebo in high-risk cardiovascular patients (62, 87). Likewise, in the Telmisartan Randomised Assessment Study in ACE-intolerant Subjects with Cardiovascular Disease (TRANSCEND), no difference was seen in AF incidence between treatment with telmisartan or placebo in patients with high-risk for cardiovascular disease (62, 88).

Aldosterone creates a substrate susceptible to atrial arrhythmias, characterized by atrial fibrosis, myocyte hypertrophy, and conduction disturbances (89). Mineralocorticoid receptor antagonists (MRAs) have emerged as potential preventive therapy for AF. A meta-analysis (14 studies, 5,332 patients, a history of heart failure in 2,866 patients) showed a reduction in new-onset AF and recurrent AF, but not post-operative AF (90). Likewise, finerenone reduced new-onset atrial fibrillation or atrial flutter in patients with chronic kidney disease and type 2 diabetes (91). However, in contrast to results in cohorts of patients with HF and a reduced ejection fraction, spironolactone does not reduce the risk of new-onset AF or AF recurrence in patients with HF and a preserved ejection fraction (HFpEF) (92).

### SGLT2 inhibitors

DECLARE-TIMI 58 (Dapagliflozin Effect on Cardiovascular Events– Thrombolysis in Myocardial Infarction 58) studied the safety and efficacy of dapagliflozin [a sodium-glucose cotransporter (SGLT2) inhibitor] vs. placebo in 17,160 type 2 diabetes mellitus patients with either multiple risk factors for atherosclerotic cardiovascular disease (*n* = 10,186) or known atherosclerotic cardiovascular disease (*n* = 6,974). Dapagliflozin decreased the incidence of *reported* AF and atrial flutter episodes adverse events in high-risk patients with type 2 diabetes mellitus. This beneficial effect was consistent regardless of the patient's prior history of AF, atherosclerotic cardiovascular disease, or HF (93). However, in an accompanying editorial, it was noted that in the absence of clear prospective definitions and systematic data collection, AF events may have been reported that should not have been, and that there could have been unreported AF episodes resulting in underestimation of the true incidence of events documented (94). In a substudy of the EMPA-REG OUTCOME (Empagliflozin, Cardiovascular Outcomes, and Mortality in Type 2 Diabetes) trial (95, 96) the HF benefits from use of the SGLT2 inhibitor empagliflozin, including early signs or symptoms of HF were consistent whether or not AF was present at baseline. In the CANVAS (Canagliflozin Cardiovascular Assessment Study) Program, there was no detectable effect of canagliflozin compared with placebo on AF for the subsets of participants with and without AF history at baseline (97).

A 2018 meta-analysis of 10,512 participants also did not find a significant association between SGLT2 inhibitor treatment and AF (98). A subsequent larger meta-analysis (including 16 trials consisting of 38,335 type 2 diabetics) found that SGLT2 inhibitors significantly reduced the combined endpoint of AF and atrial flutter (*P*  =  0.001) (99). An even larger systematic review and meta-analysis (22 trials including 52,115 patients) likewise found that SGLT2 inhibitors significantly reduced the risk of the combined endpoint of AF and atrial flutter by 18%. The authors noted that SGLT2 inhibitor treatment might be associated with a lower AF risk and contended that AF and AFL have similar clinical significance and consequences (100). We believe that this contention is not entirely correct. Although the CHA(2)DS(2)-VASc score is useful for stroke risk stratification in patients with atrial flutter, curative ablation of isthmus dependent atrial flutter is far more likely to occur than permanent elimination of atrial fibrillation (101).

Based on these (and other) reports, we remain somewhat hesitant to draw a firm conclusion about the benefits of SGLT2 inhibitors in AF prevention (102). Nevertheless, given that poor glycemic control may increase the risk of AF (see below) use of an SGLT2 inhibitor to decrease hemoglobin A1c seems reasonable.

**Figure 3:**
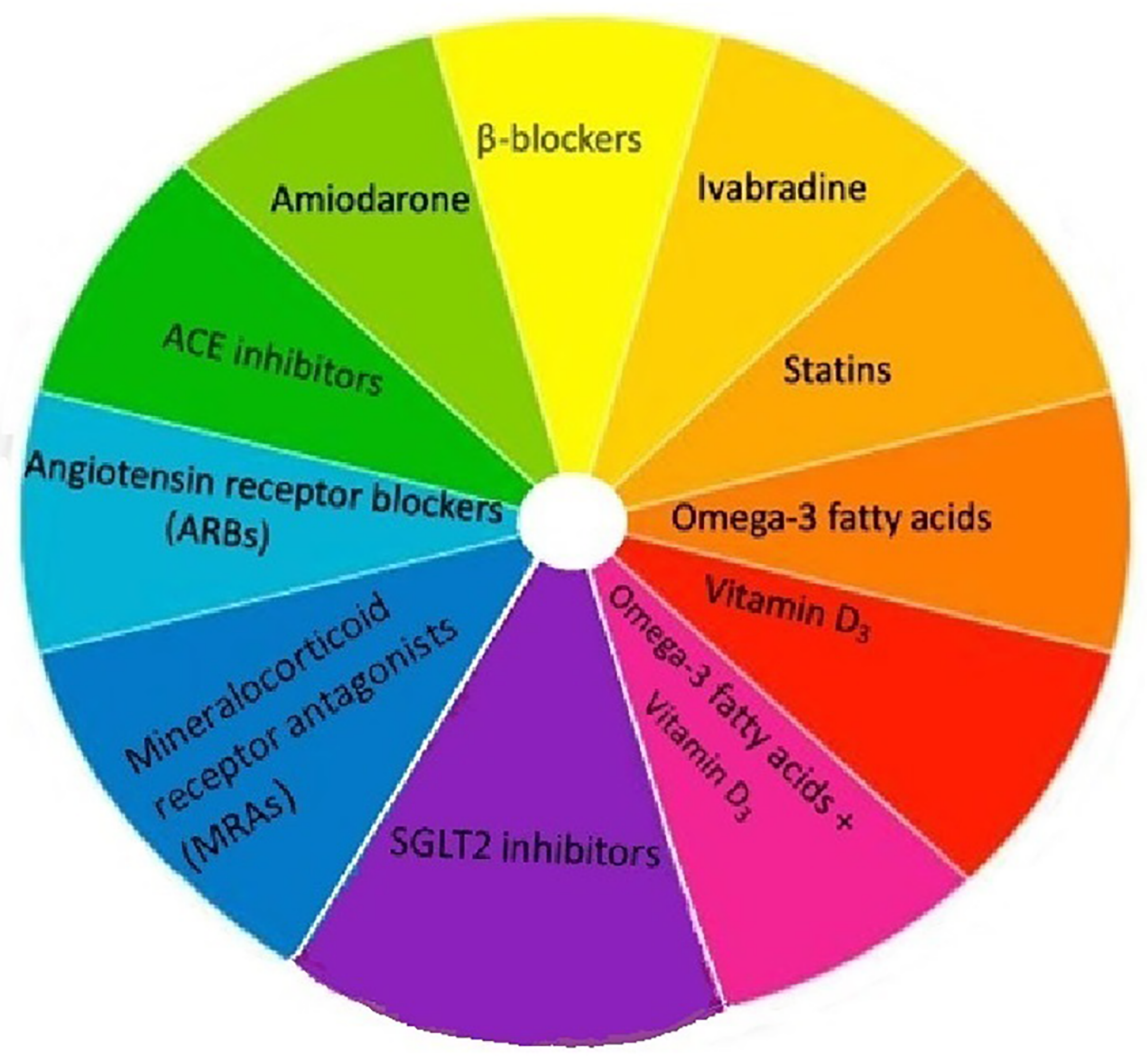
Summary of investigated pharmacological and dietary approaches for primary AF prevention.

Figure 3 provides a summary of the various pharmacological and dietary interventions that have been explored for primary AF prevention. Amiodarone, beta blockers and (to a lesser extent) ivabradine have demonstrated efficacy in prevention and management of post-operative AF. Benefits of dietary measures remain unproven. We believe, (perhaps) apart from RAAS inhibitors, pharmacological treatment for primary prevention of AF remains a work in progress.

## Risk factor modification for AF prevention and reduction

Addressing and reducing/eliminating risk factors for AF is not an easy task. It requires unique partnerships that are not easily achieved. All caregivers need to be knowledgeable and provide clear goals in a manner that is simultaneously informative, firm, and empathetic. Patients need to be introspective, cooperative and highly motivated.

## Obesity

Obesity [defined as body mass index (BMI) ≥30 kg/m^2^] is an important, strong risk factor associated with incident AF and persistent AF (103). Although obesity and elevated body mass index (BMI) predispose individuals to AF risk factors such as hypertension, diabetes mellitus, MI, left ventricular (LV) hypertrophy, left atrial enlargement, LV diastolic dysfunction, HF, and obstructive sleep apnea, they are considered an independent risk factor for AF (103).

In a study from 2002, each unit increase of BMI was associated with a significant 6% increase in the adjusted relative risks of total, ischemic, and hemorrhagic stroke (104). In 2004, Wang and colleagues observed a 4% increase in the risk of AF per 1-U increase in body mass index (BMI), at a mean follow-up of 13.7 years (105).

In a meta-analysis of five population-based cohort studies, obese individuals were noted to have a 49% increased risk of developing AF compared to nonobese individuals. In contrast, in a meta-analysis of 11 postcardiac surgery studies, obesity did not confer an increased risk of post-operative AF (106). However, a subsequent systematic review and random-effects meta-analysis of 18 observational studies revealed a modestly higher risk of post-operative AF in obese compared with nonobese patients (odds ratio: 1.12; 95% CI, 1.04–1.21; *P* = 0.002) (107).

A secondary analysis from the FANTASIIA (Atrial fibrillation: influence of the level and type of anticoagulation on the incidence of ischemic and hemorrhagic stroke) registry examined the influence of weight on the risk of adverse events in 1956 anticoagulated AF patients. In the study cohort, 358 (18.3%) had a normal body mass index, 871 (44.5%) were overweight, and 727 (37.2%) were obese. Body mass index was not independently associated with all-cause mortality, cardiovascular mortality, stroke, major bleeding, or major adverse cardiovascular events (108).

In the Atrial Fibrillation Follow-Up Investigation of Rhythm Management (AFFIRM) study, 2,492 patients with established AF were categorized and analyzed in three BMI groups. These included normal: 18.5 to <25; overweight: 25 to <30; and obese: ≥30. All-cause mortality was highest in the normal group. Cardiovascular mortality was highest in the normal group (3.1 per 100 patient-years), lowest in the overweight group (1.5 per 100 patient-years), and intermediate in the obese group (2.1 per 100 patient-years). After adjustment for baseline characteristics and risk factors, all-cause mortality did not differ significantly between the groups. However, overweight remained associated with a reduced risk of cardiovascular death (Hazard ratio 0.47, *P* = 0.002) (109).

In the ENGAGE AF-TIMI 48 trial, AF patients were randomized to anticoagulation with warfarin or edoxaban. The 21,028-patient cohort was divided into six BMI (kg/m^2^) categories (underweight (<18.5) 0.8%, normal (18.5 to <25) 21.4%, overweight (25 to <30) 37.6%, moderately obese (30 to <35) 24.8%, severely obese (35 to <40) 10.0%, and very severely obese (≥40) 5.5%). The effects of edoxaban compared to warfarin on stroke/systemic embolic events, major bleeding, and net clinical outcome were similar across BMI groups. Higher BMI was significantly and independently associated with lower risks of stroke/systemic embolic events (HR: 0.88, *P* = 0.0001), ischemic stroke/systemic embolic events (HR 0.87, *P* < 0.0001), and death (HR 0.91, *P* < 0.0001). However, higher BMI was associated with increased risks of major (HR 1.06, *P* = 0.025) and major or clinically relevant non-major bleeding (HR 1.05, *P* = 0.0007) (110).

These and other studies have reported that obesity confers a survival advantage among patients with cardiovascular disease, cancer, diabetes, respiratory disease, and renal disease, among other conditions. These studies have led to the notion of an “obesity paradox”. Adoption of this concept has led to some skepticism. In a meta-analysis of 239 prospective studies including over 10 million subjects from four continents (Asia, Australia and New Zealand, Europe, and North America), all-cause mortality was lowest for BMIs between 20 and 25 kg/m^2^ and increased to its highest levels as the BMI rose from 30 to ≤60 kg/m^2^. At the highest level of obesity, BMI 40 to ≤60 kg/m^2^, the hazard ratio was 2.76, 95% CI, 2.60–2.92. These results challenged speculation about the protective metabolic effects of increased body fat (111).

Proposed explanations for the obesity paradox include: (1) body fat aiding survival during periods of low nutrition; (2) inclusion of patients who have lost weight as a result of more severe illness among the nonobese population; (3) body mass index (BMI) poorly representing body fat; (4) inappropriate BMI cut-offs; and (5) obese people being diagnosed sooner (112).

Banack and Stokes voiced their doubts about the paradox in an editorial. They noted collider stratification bias (unmeasured confounding induced by selection bias) as a possible reason for the apparent paradox. In addition, they suggested (as noted above) that a possible explanation is the potential for bias due to illness related weight loss (113). Sperrin and colleagues have contended that collider bias may partially explain the obesity paradox but is unlikely to be the main explanation for a reverse direction of an association to a true causal relationship (112).

Like Sperrin et al., we believe that because obesity is a risk factor for AF and other comorbidities, the obesity paradox challenges common sense. Based on the conflicting data described above, we believe skepticism has a role in leading to clarification of this paradox, but we cannot deny that this paradox may be true (112).

A mendelian randomization analysis of over 50,000 individuals of European ancestry revealed genetic variants associated with a high BMI correlated with AF incidence, suggesting a causal relationship between BMI and AF (114). Targeting at least a 10% reduction in weight has been suggested to effect reductions in AF burden. BMI reduction to <27 kg/m^2^ has been advocated (115, 116). However, BMI has limitations. The risk of AF as determined in a large cohort analysis appeared to be driven by low lean body mass rather than BMI alone or anthropomorphic obesity patterns such as hip or waist circumference (117).

The results above are summarized in Table 2. We believe that targeting a 10% weight reduction is a reasonable goal. The obesity paradox is thought provoking and is a puzzle that needs to be “solved”. We look forward to additional clarification of this issue.

**Table 2:** Obesity and AF.

1st Author Ref. # Year	Cases/participants	Age, years	Follow-up, years (unless otherwise indicated)	Study type	Key findings/messages
Wang et al. (105) 2004	526/5,282	57 ± 13	13.7	Prospective, community-based observational cohort	• Body mass index (BMI) analyzed as continuous and categorical variables (normal <25.0; overweight, 25.0 to <30.0; and obese, ≥30.0). • Age-adjusted incidence rates for AF increased across the 3 BMI categories. • A 4% increase in AF risk per 1-unit increase in BMI was observed in men [95% confidence interval (CI), 1%–7%; *P* = .02] and women (95% CI, 1%–7%; *P* = .009). Adjusted AF hazard ratios associated with obesity were (HR:1.52; 95% CI, 1.09–2.13; *P* = .02) and (HR:1.46; 95% CI, 1.03–2.07; *P* = .03) for men and women, respectively, when compared with individuals with normal BMI. • The excess AF risk associated with obesity appeared to be mediated by left atrial dilatation.
Wanahita et al. (106) 2008	Normal weight: 407/30,597 Overweight: 614/29,429 Obese: 358/11,139	Population-based cohorts: 56 ± 2 Post-cardiac surgery: 64 ± 2	4.7 to 25.2 years in the population-based cohort studies. Post-cardiac surgery during index hospitalization	Meta-analysis	• Based on the population-based cohort studies, obese individuals had a 49% increased risk of developing AF. • In the post-cardiac surgery cohort, obesity did not result in an increased risk of developing AF
Bertomeu-Gonzalez et al. (108) 2020	727/1,956	73.8 ± 9.4	1,070 days	Observational, multicenter, and prospective registry	• In this patient cohort anticoagulated for AF, obesity was highly prevalent and was associated with more comorbidities, but not with poor prognosis (all-cause mortality, cardiovascular mortality, stroke, major bleeding, or major adverse cardiovascular events).
Ardestani et al. (109) 2010	890/2,492	Normal BMI: 72.4 ± 7.1 Overweight: 72.4 ± 7.1 Obese: 66.4 ± 8.4	Mean follow-up was 3.5 (range: 0–6 years)	Affirm sub-study	• There was no significant difference in the rate of stroke. • The observed cardiovascular death rate was highest among patients in the normal BMI group. • The total observed death rate was significantly higher among patients in the normal BMI group. • Obesity (even severe and extreme obesity) was not associated with a higher risk of death. • Overweight was independently associated with a substantially reduced risk of death from cardiovascular causes.
Boriani et al. (110) 2019	Underweight: 177/21,028 Normal weight: 4,491/21,028 Overweight: 7903/21,028 Moderately obese: 5,209/21,028 Severely obese: 2,099/21,028 Very severely obese: 1,149/21,028	Underweight: 73 Normal weight: 75 Overweight: 75 Moderately obese: 71 Severely obese: 68 Very severely obese: 64	Median 2.8	ENGAGE AF-TIMI 48 trial sub-study	• Higher BMI was significantly and independently associated with lower risks of stroke/systemic embolic events (SEE) (HR 0.88, 95% CI, 0.82–0.94, *P* = 0.0001); ischemic stroke/SEE (HR 0.87; 95% CI, 0.81–0.93, *P* < 0.0001), and of death (HR 0.91; 95% CI, 0.87–0.95, *P* = 0.0001). • However, increasing BMI was independently associated with a greater risk of major bleeding (HR 1.06; 95% CI, 1.01–1.12, *P* = 0.025) and of major or clinically relevant non-major bleeding (HR 1.05; 95% CI, 1.02–1.08, *P* = 0.0007).
Chatterjee (114) 2017	4178/51,646	Ranged from 49 to 76	7.4–19.2	Meta-analysis OSA patients who received CPAP therapy had a risk of recurrent AF similar to that of the non-OSA patients.	• Used 2 genetic tools*:* 1) *FTO* single-nucleotide polymorphism (SNP; rs1558902) locus that demonstrated the strongest association with BMI in previous genetic analyses. 2) A weighted genetic score (BMI gene score) using 39 single-nucleotide polymorphisms. • Increasing BMI was uniformly associated with a significantly increased risk of incident AF. • After adjustment for potential confounders the hazard ratio for the association was (HR: 1.04; 95% CI, 1.03–1.05; *P *< 0.001). • Genetic variants associated with increasing BMI were significantly associated with incident AF. Increments in risk ranged from 3% to 6% per 1 kg/m^2^ increase in BMI.
Pathak (116) 2015	355/1,415	Weight loss by group ≥10% 65 ± 11 3–9% 63 ± 11 <3% 63 ± 11	Months ≥10%: 48.4 ± 18.2 3–9%: 46.0 ± 16.7, <3%: 48.3 ± 18.4	Cohort study	• Systolic BP decreased the most in the ≥10% group. • For diabetics, glycemic control improved with more weight loss. • Greater reduction in low-density cholesterol, triglycerides, and total cholesterol levels was seen in the 10% group (*P* < 0.001). • AF frequency, duration, symptoms, and symptom severity were improved in groups with weight loss ≥3% compared with <3% group. • Left atrial volume indexed for body surface area decreased significantly with ≥3% weight loss.

Weight loss is recommended to treat obstructive sleep apnea (another AF risk factor which is discussed in more detail below).

## Physical inactivity

A sedentary lifestyle is associated with higher AF risk (103, 118). Inactivity, in turn, increases the risk of other AF risk factors, including hypertension, obesity, and diabetes. It is also associated with obesity and obstructive apnea. Moderate, regular, physical activity is a cornerstone of a healthy lifestyle (119). It is inversely and independently associated with clinical AF incidence and progression. A number of studies indicate beneficial effects on AF prevention and/or progression in individuals pursuing regular physical actvity (118–122).

In the AusDiab study, after adjustments for associations between prevalent AF and baseline characteristics, the prevalence ratio of AF was 2.1 when sedentary individuals were compared to those deemed to have sufficient physical activity (120). Likewise, Calvo et al. found that that compared to sedentary individuals, those with a lifetime history of <2,000 h of high-intensity training had significant protection against lone AF [OR 0.38 (0.12–0.98)] (121). In the Cardiovascular Health Study, Mozaffarian and associates followed 5,446 adults ≥65 years old for 12 years and documented 1,061 new AF cases (incidence rate 22.4 cases per 1,000 person-years). Compared to individuals without regular exercise, moderate-intensity exercise resulted in a 28% lower risk of AF, however, individuals with high-intensity exercise did not have significantly lower risk than those who did not exercise regularly (122). In a prospective study of 2,869 patients with paroxysmal or persistent AF, after a median follow up of 3 years, regular physical activity was inversely predictive of progression from paroxysmal to non-paroxysmal or persistent AF to permanent AF (HR, 0.80; 95% CI, 0.66–0.98) (119).

In contrast, extreme levels of physical activity may be associated with a higher AF risk (103, 118) and have paradoxically been associated with increased AF burden. Calvo and associates noted that AF risk increased with ≥2,000 h lifetime-accumulated high-intensity training [OR 3.88 (1.55–9.73)] (121).

In a meta-analysis 655 athletes and 895 controls were compared. Mean age was 51 ± 9 years and 93% were men. There were 147 (23%) vs. 116 (12.5%) cases of AF among athletes compared with controls. The overall risk of AF was significantly higher in athletes than in controls with odds ratio (95% confidence interval) = 5.29 (3.57–7.85), *P* = 0.0001 (123).

Athletes who engage in endurance sports such as runners, cyclists and skiers are more prone to AF than other athletes (124). The mechanisms by which exercise training increases AF risk are complex and may include atrial dilation, adrenergic activation, vagal tone, chronic inflammation, pulmonary foci and interstitial fibrosis, resulting from excessive strain through augmented cardiac output and atrial stretch (125, 126).

High-intensity interval training (HIIT) involves performing repeated periods of intense exercise interspersed with low-intensity exercise or periods of rest with varied recovery times. Exercise periods may range from 5 seconds to 8 minutes long with recovery periods varying in length and total exercise duration lasting between 20 and 60 minutes (127). In a recent randomized clinical trial including 86 individuals with AF, HIIT was as efficacious as moderate to vigorous intensity continuous training (MICT) in improving functional capacity and general quality of life, despite a substantially lower total exercise volume. HIIT was also as effective as MICT in improving disease-specific resting heart rate, physical activity levels and quality of life (128). HIIT improves fitness and cardiac function, however its impact on LA structural and electrical remodeling as well as AF burden is not completely understood. It should be noted that in endurance-trained men, HITT resulted in left atrial enlargement. The extent of LA dimensional remodeling in highly trained athletes may be relevant, and absolute LA size can overlap atrial dilation observed in patients with cardiac disease (129). This implies, but does not prove, the potential to develop AF.

Regular aerobic exercise at the levels recommended by the 2018 Physical Activity Guidelines Advisory Committee (150 min/week of moderate-intensity or 75 min/week of vigorous-intensity aerobic exercise) does not increase AF risk and may reduce the risk of new-onset AF (130).

Hence, regular moderate exercise is likely to help prevent AF incidence and progression. In contrast, high intensity endurance athletics increase the risk of incident AF. Whether HIIT provides protection from AF is unknown.

Interestingly, a relatively small single-center study enrolled 49 patients with symptomatic paroxysmal AF who underwent an initial 3-month control noninterventional observation period followed by an interventional phase of twice-weekly 60-min yoga training (a combination of structured physical exercises, breathing techniques, and meditation) for next 3 months. Yoga significantly reduced the number of symptomatic and asymptomatic AF episodes from the end of control phase to the end of intervention phase. Although the precise mechanisms underlying yoga's AF benefits were unknown, the authors speculated that yoga may prevent initiation and perpetuation of AF *via* one or more of the following mechanisms: increasing the baseline parasympathetic tone, suppressing extreme sympathetic/parasympathetic fluctuations, and decreasing arrhythmia progression by preventing or minimizing atrial remodeling (131).

We think that exercise has important clinical benefits. We agree with Shakespeare that “Nothing comes from doing nothing” and wonder “Why then, can one desire too much of a good thing?”. Table 3 summarizes key components of the data provided above.

**Table 3:** Physical inactivity.

1st Author Ref. # Year	Cases/participants	Age, years	Follow-up, years (unless otherwise indicated)	Study type	Key findings/messages
Diouf et al. (120) 2015	90/8,273	56.6 ± 11.7	3.6 years to 6.0 years (Average 5.0 years)	National population-based sample	• AF prevalence was higher among individuals classified as “sedentary” compared to those classified as having “sufficient physical activity” (prevalence ratio 2.1; 95% CI, 1.2–3.6).
Calvo et al. (121) 2016	115/172	46 ± 10	Accumulated lifetime activity	Prospective, observational, cross-sectional study	• AF risk increased with an accumulated lifetime endurance sport activity ≥2,000 h compared with sedentary individuals (OR: 3.88; 95% CI, 1.55 –9.73). A history of <2,000 h of high-intensity training protected against AF when compared with sedentary individuals (OR 0.38; 95% CI, 0.12–0.98). • After adjusting for cumulative hours of exercise, endurance sports carried a higher AF risk in comparison to team sports (OR 5.68; 95% CI, 1.72 –18.7).
Mozaffarian et al. (122) 2008	1,061/5,446	72.8 ± 5.6	12	Meta-analysis	• Light to moderate physical activities, particularly leisure-time activity and walking, were associated with significantly lower AF incidence in older (age >65) adults. • Individuals with moderate intensity exercise had 28% lower AF risk, but those with high intensity exercise did not have significantly lower risk than those with no regular exercise.
Abdulla et al. (123) 2009	Athletes: 147/655 Controls: 116/895	51 ± 9	1–28	Systematic review and meta-analysis	• Atrial flutter cases were included under the designation AF. • The overall risk of AF was significantly higher in athletes compared to controls (OR: 5.29; 95% CI, 3.57–7.85, *P* = 0.0001).
Newman et al. (125) 2016	6,816/70,478		≥ 2 years	Systematic review and meta-analysis	• In athletes and non-athletes without CVD risk factors, athletes had a significantly greater relative risk of AF (OR: 3.66; 95% CI, 2.28 to 5.88, *P* < 0.001). Younger athletes (OR: 3.60; 95% CI, 2.09 to 6.29, *P* < 0.001) had a significantly higher relative risk of AF than older athletes (OR: 1.76; 95% CI, 0.97 to 3.21, *P* = 0.065). • Mixed (both genders vs. hybrid??) sports conferred a higher risk of AF compared to endurance sports. A definition of mixed sports was not provided.
Reed et al. (128) 2022	86/94 participated	69 ± 7	12 weeks	Randomized clinical trial	• Participants had persistent or permanent AF. • Compared the effects of 12 weeks of High-intensity interval training (HIIT) to moderate to vigorous intensity continuous training (MICT)-based cardiovascular rehabilitation (CR) on functional capacity and general QOL in patients with persistent and permanent AF. • No significant differences in improvements in functional capacity (6-minute walk test). • No significant difference in improvement of disease specific QOL between protocols. • Twice-weekly 23-minute HIIT was as efficacious as twice-weekly 60-minute cardiac rehabilitation in improving functional capacity, general and disease-specific QOL, resting HR, and physical activity.
Mahjoub et al. (129) 2019	8/17: 85% maximal aerobic power (HIIT_85_). 9/17: 115% maximal aerobic power (HIIT_115_).	N/A extension of a study where the mean age was 27 ± 7	Study duration 6 weeks	Case series	6 weeks of high-intensity interval training in endurance athletes increases left atrial volumes irrespective of training intensity (85 or 115% maximal aerobic power).

## Hypertension

Because of hypertension's high prevalence, this risk factor is associated with the highest attributable risk for AF development (21, 132). It has been estimated that hypertension is responsible for 14% of all AF cases and it is the most significant population-attributable (the proportional reduction in population disease if exposure was reduced to an alternative ideal scenario) AF risk factor (133, 134). Hypertension was present in >70% of AF patients in epidemiological studies (133, 135, 136) and recent AF real-world registries (133, 137–139), and in 49%–86% of patients in randomized AF trials (133, 140, 141).

As noted above, employing angiotensin-converting enzyme inhibitors and angiotensin receptor blockers as antihypertension therapy has yielded inconsistent results with regard to AF primary prevention. In contrast, mineralocorticoid receptor antagonist treatment was associated with reduced AF risk and recurrence. The Substrate Modification With Aggressive Blood Pressure Control study (SMAC AF) was a randomized, open-label trial of tight BP control compared with standard care in patients undergoing AF ablation. When moderate hypertension was managed as an isolated risk factor, no difference in arrhythmia control was observed (142). In contrast, two studies randomized patients (27 and 76) with severe resistant hypertension and symptomatic AF to ablation (pulmonary vein isolation) with or without renal sympathetic denervation. In the smaller of these two studies, weekly ECGs were obtained for the first month, and 24-h Holter recordings were performed at 3, 6, 9, and 12 months. In the latter study, all patients received an implantable loop recorder on the day of their ablation. Renal sympathetic denervation was associated with a significant reduction in BP and AF burden at 12 months (143, 144). The ASAF trial is ongoing and will attempt to further elucidate whether renal denervation plus pulmonary vein isolation reduces AF recurrence. The investigators aim to randomize 138 hypertensive patients with AF and signs of sympathetic overdrive in a 1:1 fashion to pulmonary vein isolation alone vs. pulmonary vein isolation plus renal sympathetic denervation (145).

Management of hypertension is a pivotal part of AF amelioration. Addressing the *renin-angiotensin-aldosterone system is a very important part of this goal. As noted above,* aldosterone creates a substrate susceptible to atrial arrhythmias (89) and mineralocorticoid receptor antagonists (MRAs) have emerged as potential preventive therapy for AF (90). Table 4 below, summarizes some of the key issues associated with AF and hypertension.

**Table 4:** Hypertension and AF.

1st Author Ref. # Year	Cases/participants	Age, years	Follow-up, years	Study type	Key findings/message
Lip et al. (133) 2017	-----	-----	-----	Consensus statement	AF should be considered as a manifestation of hypertensive heart disease, and hypertension management should be optimized. Stroke prevention is central to the management of AF patients; detection of hypertension and good blood pressure control should be achieved to minimize the risk of stroke/thromboembolism, as well as the bleeding risk on antithrombotic therapy.
Kannel et al. (134) 1998	562/4,731	50–94	38	Framingham Heart Study	Hypertension is responsible for 14% of all AF cases. Men have a 1.5-fold greater risk of developing AF than women. AF prevalence doubles with each decade of age, from 0.5% at age 50–59 years to almost 9% at age 80–89 years. AF is becoming more prevalent.
Kakkar et al. (135) 2013	8,249/10,614	70.2 ± 11.2	2	International observational study	77.8% of the participants had hypertension. Worldwide observational data on non-valvular AF, collected at the end of the vitamin K antagonist-only era, demonstrated that these drugs are frequently not being used per stroke risk scores and guidelines. Mean CHA_2_DS_2_-VASc score was 3.2 ± 1.6, and 84.4% (8,957/10,607) had a score ≥2. 40.7% of the patients with a CHA_2_DS_2_-VASc score ≥2 did not receive guideline-recommended anticoagulant prophylaxis.
Chiang CE et al.. (136) 2012	10,546/10,546	Paroxysmal 64.7 ± 12.4 Persistent 66.0 ± 11.8 Permanent 68.3 ± 11.8	-----	International survey	2,606 (26.5%) had paroxysmal, 2,341 (23.8%) persistent, and 4,869 (49.6%) permanent AF. Hypertension was the most common cardiovascular risk factor (paroxysmal AF 74.6%, persistent AF 73.2%, permanent AF 71.6%). Over half of all patients with AF qualified for oral anticoagulants, but they were used inadequately.
Lip GY et al. (137) 2014	2,162/3,049	68.8	3	Pilot General Registry	Hypertension present in 70.9%. Oral anticoagulants were used in 80% overall, most often vitamin K antagonists (71.6%), with novel OACs being used in 8.4%. Oral anticoagulants were used in 56.4% of CHA_2_DS_2_-VASc = 0. Oral anticoagulant use was only 66.7% in CHA_2_DS_2_-VASc score 9.
Potpara T et al. (139) 2016	2,108/2,663	69.1 ± 10.9	0.27	BALKAN-AF Survey	Hypertension present in 79.2%. Overall, 73.6% received oral anticoagulants (17.2% DOACs). 56.5% of very low risk (CHA_2_DS_2_-VASc = 0 [males], or 1 [females]) subjects received OAC.
Hohnloser S et al. (140) 2000	123/252	Diltiazem group: 61 ± 9 Amiodarone group 60 ± 10	1	Randomized Trial	125 patients randomized to amiodarone; 127 to diltiazem. Hypertension present in 49%. No difference in symptoms or quality of life between the groups. Amiodarone restored sinus rhythm more often (56% vs. 10%, *P* < 0·001) but caused more side effects (*P* = 0·011).
Connolly S (141) 2011	4,837/5,599	70	1.1	Randomized Trial	Hypertension was present in 86% of patients. Designed to compare the efficacy and safety of apixaban, 5 mg twice daily, vs. aspirin, at a dose of 81 to 324 mg daily. Risk of stroke or system embolism lower with apixaban (HR: 0.45; 95% CI, 0.32–0.62; *P* < 0.001). No difference in risk of major bleeding.

## AF and chronic kidney disease

AF and chronic kidney disease (CKD) are linked by common predispositions such as hypertension, diabetes mellitus, and coronary artery disease. The presence of CKD increases the risk of AF incidence while the presence of AF is associated with development and progression of CKD (bidirectional relationship). In CKD, the overall prevalence of AF is about 2- to 3- fold greater than the estimate of 2%–4% in the general population (146).

In a 2010 report, the [prospective] Chronic Renal Insufficiency Cohort (CRIC) study enrolled 3,267 adult participants. AF was present in 18% of the study participants and >25% of those ≥70 years old (147). In a 2016 report, among 3,091. participants without AF at entry, 172 (5.6%) developed incident AF during follow-up. During a mean follow-up of 5.9 years, 43 patients had end stage renal disease (ESRD) that occurred after the onset of incident AF (11.8/100 person-years) compared with 581 patients without incident atrial fibrillation (3.4/100 person-years). Incident AF was associated with a substantially greater rate of ESRD (hazard ratio, 3.2; 95% confidence interval, 1.9 to 5.2). This association was consistent across subgroups by age, sex, race, diabetes status, and baseline eGFR (148).

Laukkanen and associates performed a prospective study designed to evaluate whether cystatin C- and creatinine-based estimation of glomerular filtration rate (eGFRcys and eGFRcreat) and urinary albumin/creatinine ratio (ACR) were associated with a risk of AF. The study population included 1,840 subjects between the ages 61–82 years. During a follow-up (median 3.7 years), 159 (8.6%) incident AF cases occurred. Reduced eGFR and albuminuria were associated with an increased risk of atrial fibrillation. In subjects with eGFRcys of 15–59 ml/min per 1.73 m^2^ compared to those with ≥90 ml/min per 1.73 m^2^, AF risk was increased [hazard ratio 2.74, 95% confidence interval (CI) 1.56–4.81, *P* < 0.001]. Comparing participants defined by their eGFRcreat levels produced similar results (hazard ratio 2.41, CI, 1.09–5.30, *P* = 0.029). Individuals with an ACR ≥300 mg/g were compared to those with an ACR < 30 mg/g and, likewise, had an increased incidence of AF (hazard ratio 2.16, CI, 1.35–2.82, *P* < 0.001) (149). Likewise, in a meta-analysis of 3 cohorts (16,769 participants), reduced eGFR and elevated urine albumin-to-creatinine ratio were significantly associated in a stepwise inverse pattern where decreasing function posed a greater risk of incident AF (150).

Amongst 116,184 adults with CKD enrolled in The Stockholm CREAtinine Measurements (SCREAM) Project, 13,412 (12%) developed clinically apparent AF during a mean follow-up of 3.9 years. AF incidence increased across lower eGFR strata: from 29.4 to 46.3 atrial fibrillations per 1,000 person-years in subjects with eGFR = 45–60 and <30 ml/min per 1.73 m^2^. Incident AF was associated with higher risk of stroke which was similar across all eGFR strata (hazard ratio, 2.00; 95% confidence interval, 1.88 to 2.14) and death (hazard ratio, 1.76; 95% confidence interval, 1.71 to 1.82). This was attributed to both ischemic stroke (hazard ratio, 2.11; 95% confidence interval, 1.96 to 2.28) and intracranial bleeds (hazard ratio, 1.64; 95% confidence interval, 1.42–1.90) (151).

Watanabe et al. performed a prospective community-based observational cohort study including 235,818 Japanese subjects. During 5.9 ± 2.4 years of follow up 2,947 subjects (1.3%) developed AF. Baseline serum creatinine and estimated glomerular filtration rate (GFR) were associated with a subsequent risk of AF. The hazard ratios [HRs (95% CI)] for AF were 1.32 (1.08–1.62) and 1.57 (0.89–2.77) for GFRs 30 to 59 and <30 ml/min per 1.73 m^2^, respectively. During follow-up, 7,791 subjects (3.3%) developed renal dysfunction (GFR <60 ml/min per 1.73 m^2^ and 11,307 subjects (4.9%) developed proteinuria. AF at entry was associated with development of both renal dysfunction (HRs [95% CI], 1.77 [1.50–2.10]) and proteinuria (HR [95% CI], 2.20 [1.92–2.52]) (152).

In the REGARDS study, the association of CKD with ECG-detected AF was evaluated in 26,917 African-American and white United States adults ≥45 years old. Patients were grouped in stages according to renal function: no CKD (eGFR ≥60 ml/min/1.73 m^2^ without albuminuria, *n* = 21,081), stage 1 to 2 CKD (eGFR ≥60 ml/min/1.73 m^2^ with albuminuria *n* = 2,938), stage 3 CKD (eGFR 30 to 59 ml/min/1.73 m^2^, *n* = 2,683) and stage 4 to 5 CKD (eGFR <30 ml/min/1.73 m^2^, *n* = 215). The AF prevalence was 1.0% among adults without CKD, and 2.8%, 2.7% and 4.2% among adults with stage 1–2, stage 3 and stage 4–5 CKD, respectively (153).

The EurObservational Research Programme AF General Pilot Registry (EORP-AF) assessed 1-year outcomes in 2,398 patients with AF in relation to kidney function. Glomerular filtration rate (eGFR) was an independent predictor of stroke/TIA or death, with elevated odds ratios concordant with the severity of renal impairment: eGFR < 30 ml/min/1.73 m^2^ [OR 3.641, 95% CI, 1.572–8.433, *P* < 0.0001], 30–49 ml/min/1.73 m^2^ [OR 3.303, 95% CI, 1.740–6.270, *P* = 0.0026] or 50–79 ml/min/1.73 m^2^ [OR 2.094, 95% CI, 1.194–3.672, *P* = 0.0003] (154).

Fauchier and colleagues examined the impact of declining eGFR in 2,653 AF patients with information on worsening of kidney function during a mean of 1,499 days of follow-up. Patients were divided into 4 quartiles based on the slope of their worsening renal function. There was an increased risk of stroke/thromboembolism when the decline in eGFR was more marked (HR 1.226, 95% CI, 1.087–1.381 for each change of quartile) and the risk was markedly increased when patients in the 4th quartile were compared to other patient groups (HR 1.803, 95% CI, 1.367–2.378). Likewise, there was an increased bleeding risk when the decline in eGFR was more marked (HR 1.184, 95% CI, 1.071–1.308 for each change of quartile) and the risk was particularly increased when patients in the 4th quartile were compared to other patients (HR 1.582, 95% CI, 1.245–2.010). Stroke/thromboembolism and all-cause mortality rates were lower in individuals on oral anticoagulation (OAC), compared with those not on OAC. The effect was not significantly affected by worsening eGFR quartiles. Bleeding rates were higher in individuals on OAC, compared with non-anticoagulated patients. This effect was also not significantly affected by eGFR worsening quartiles (155).

Taken together these findings are consistent with the bidirectional relationship between AF and CKD. The impact of advancing renal disease on AF incidence (AKA the dose-response) and adverse AF related outcomes is clear. Table 5 below summarizes studies that emphasize the bidirectional influences between AF and CKD (146, 148–152, 156–160).

**Table 5:** Bidirectional relationship between AF and CKD.

Study (year)	Population	*n*	Groups	Follow-up (years)	Finding(s)
Guo et al. (2019) (156)	Chinese adults	88,312	CKD vs. non-CKD	NA	Increased prevalence of AF by four-fold in CKD; dose–response relation between incident AF and worsening CKD
Carrero et al. (2018) (151)	eGFR <60 without AF	116,184	eGFR 60–89; 45–59; 30–44; and <30	3.9	Dose–response relation between incident AF and worsening eGFR; eGFR <30 was associated with a 1.6-fold increased risk of incident AF (reference eGFR 45–60)
Marcos et al. (2017) (157)	Population-based cohort, enriched by those with albuminuria	8,265	Creatinine, eGFR, cystatin C and urine albumin excretion as continuous variables	9.8	No association between incidence of AF and markers of renal function (creatinine, eGFR, and cystatin C); dose–response relation between incident AF and urine albumin excretion
Laukkanen et al. (2016) (149)	Population-based cohort	1,840	eGFR ≥90 vs. eGFR 60–89 vs. eGFR 15–59; macroalbuminuria vs. no albuminuria	3.7	eGFR 15–59 was associated with a 2.7-fold increased risk of incident AF (reference eGFR ≥90); higher incidence of AF with macroalbuminuria
Alonso et al. (2011) (158)	Population-based cohort	10,328	eGFR ≥90 vs. eGFR 60–80 vs. eGFR 30–59 vs. eGFR 15–29; macroalbuminuria vs. microalbuminuria vs. no albuminuria	10.1	Dose–response relation between incident AF and worsening eGFR; risk of incident AF increased even with mild renal dysfunction; eGFR 15–29 was associated with 3.2-fold increased risk of incident AF (reference eGFR ≥90); higher incidence of AF with albuminuria
Deo et al. (2010) (159)	Ambulatory elderly patients	4,663	Cystatin C quartiles; eGFR ≥60 vs. eGFR <60	7.4	No association between incidence or prevalence of AF and eGFR; two highest quartiles of cystatin C levels were each associated with a 1.5-fold increased risk of incident AF (reference Quartile 1); no association between prevalence of AF and cystatin C levels
Iguchi et al. (2008) (160)	Population-based cohort	41,417	eGFR tertiles	NA	Higher prevalence of AF with decreasing eGFR tertiles; OR 1.91 (95% CI of 1.54–2.38) in the lowest tertile compared to the highest tertile
Bansal et al. (2016) (148)	CKD without AF and ESRD	3,091	Incident AF vs. no incident AF	5.9	Incident AF in CKD was associated with a 3.2-fold increased risk of developing ESRD requiring RRT or kidney transplant
Bansal et al. (2017) (150)	Meta-analysis of 3 prospective cohorts across categories of decreasing GFR and increasing urine albumin-to-creatinine ratio	16,769	eGFR was modeled continuously (per 20 ml/min per 1.73 m^2^* lower eGFR) as well as in categories (90, 60–89, 45–59, 30–44, and <30 ml/min per 1.73 m^2^*). UACR was modeled continuously and in clinically based categories: <15, 15–29, 30–299, and >300 mg/g.	JHS 8.5 ± 2.7 MESA 10 ± 3.0 CHS 12.5 ± 7.1	Reduced eGFR and elevated urine albumin-to-creatinine ratio were significantly associated with greater risk of incident AF. There was a stepwise increase in the adjusted risk of incident AF across categories of decreasing eGFR. The greatest risk was among participants with an eGFR <30 ml/min per 1.73 m^2^. Meta-analysis of JHS and MESA, revealed a stepwise association between higher UACR and risk of incident AF, with the greatest risk among participants with UACR 300 mg/g (hazard ratio, 1.76; 95% confidence interval, 1.18 to 2.62) compared with those with UACR<15 mg/g.
Watanabe et al. (2009) (152)	Population-based cohort	235,818	Incident AF vs. no incident AF; eGFR ≥60 vs. eGFR 30–59 vs. eGFR <30	5.9	Incident AF was associated with a three-fold increased risk developing CKD; higher incidence of AF with worsening eGFR

AF, atrial fibrillation; CI, confidence interval; CKD, chronic kidney disease; eGFR, estimated glomerular filtration rate; ESRD, end-stage renal disease; OR, odds ratio; RRT, renal replacement therapy; UACR, urine albumin-to-creatinine ratio; JHS, Jackson Heart Study, MESA, Multi-Ethnic Study of Atherosclerosis; CHS, Cardiovascular Health Study.

Adapted from reference (146) with permission.

## AF and diabetes mellitus (DM)

DM may predispose to structural, electrical, and autonomic changes and is associated with a higher risk of AF (161). Data from the Framingham Heart Study revealed that men and women with diabetes mellitus had a 40% and 60% increased risk of developing AF. Evidence suggests that AF development is associated with poor glycemic control and may be related to longer diabetes mellitus duration (21, 103).

In a sample of 3,014 patients from the Non-invasive Monitoring for Early Detection of Atrial Fibrillation (NOMED-AF) study, Polish participants were divided into two groups based on the presence or absence of diabetes mellitus. In the diabetes group, none had type 1, therefore, the analyses comprised solely type 2 diabetics.

AF was noted in 22.6% of the study population. AF prevalence was significantly higher in diabetic individuals compared to those without diabetes (25%; 95% CI, 22.5%–27.8% vs. 17%; 95% CI, 15.4%–18.5% respectively, *P*  <  0.001). Asymptomatic (“silent”) AF (SAF) was more common among the diabetic group (9%; 95% CI, 7.9–11.4 vs. 7%; 95% CI, 5.6–7.5, *P*  <  0.001). Likewise, persistent/permanent AF was more common in the diabetic group (12.2%; 95% CI, 10.3–14.3 vs. 6.9%; 95% CI, 5.9–8.1, *P*  <  0.001).

The diabetic group had a significantly higher prevalence of each of the following comorbidities: acute coronary syndrome, peripheral artery disease, and hypertension. Additionally, they were less physically active and significantly more obese. The most significant limitation of this study included the absence of differentiation between atrial fibrillation and atrial flutter (162).

In a 2011 meta-analysis involving information on 108,703 AF cases among 1,686,097 individuals from 7 prospective cohort and 4 case-control studies, DM was associated with an overall increase of nearly 40% in the risk of AF after correcting for the presence of publication bias (163). A 2018 Swedish study that included 71,483 adults with type 1 and type 2 DM found that both were associated with increased risk of major cardiovascular disease outcomes. However, only insulin-dependent type 2 DM of ≥20 years duration was associated with increased risk of AF (164).

Dublin et al. noted that the risk of AF increased 3%/year in pharmacologically treated diabetics. Compared to non-diabetics, the adjusted odds ratios for AF in treated diabetics with an average hemoglobin A1c ≤7 was 1.06 (95% CI, 0.74–1.51); for A1c >7 but ≤8, 1.48 (95% CI, 1.09–2.01); for A1c >8 but ≤9, 1.46 (95% CI, 1.02–2.08); and for A1c >9, 1.96 (95% CI, 1.22–3.14) (165).

Two large Taiwanese studies demonstrated that aggressive blood sugar control was associated with a decreased risk of AF incidence and recurrence. A population-based study (645,710 subjects) revealed that using metformin was associated with a 19% lower risk of AF over 13 years compared with controls (166). In 12,605 patients with non-insulin dependent DM, treatment with a thiazolidinedione (rosiglitazone) over a period of 5-years, after adjustment for age and comorbidities, was associated with reduced risk of AF occurrence by approximately 30% (167).

Saliba et al. Investigated an Israeli cohort of 37,358 individuals with AF. Diabetes mellitus was noted in 11,713 (30.9%). The remaining 26,182 (69.1%) were not diabetic. The diabetic patients were divided into four group according to their HgbA1c: 1) <6.35%; 2) 6.35%–6.90%; 3) >6.90%–7.70% 4) >7.70%, After adjusting for CHA_2_DS_2_-VASc score risk factors, TIA and stroke were significantly more common in both group 3 (*P* < .001) and group 4 (*P* < .001) (168).

In contrast, data from 1933 diabetic patients in the ATRIA (Anticoagulation and Risk Factors in Atrial Fibrillation) cohort of AF patients revealed that 46% had a HbA1c < 7.0%, 36% between 7.0% and 8.9%, and 19% ≥9.0% at baseline. Moderate (7.0–8.9%) or poor (≥9.0%) glycemic control was not associated with a significantly increased ischemic stroke rate compared with patients who had HbA1c < 7.0%. A diabetes duration of ≥3 years was associated with an increased ischemic stroke rate compared with a duration <3 years (adjusted hazard ratio [HR]: 1.74, 95% confidence interval [CI], 1.10 to 2.76) (169).

Among a cohort of 135,222 Danish patients with AF, 12.4% were identified as having both AF and diabetes mellitus. The diabetic patients were divided (and analyzed) according to diabetes duration into 4 groups (0–4 years, 5–9 years, 10–14 years and ≥15 years). The thromboembolic risk was lowest in the 0 to 4 years duration category (hazard ratio, 1.11; 95% confidence interval, 1.03–1.20), and highest in the longest duration category of ≥15 years (hazard ratio, 1.48; 95% confidence interval, 1.29–1.70) (170).

Most of the studies discussed above were racially and ethnically homogenous. It is important to recognize that large numbers of studies have demonstrated that AF is less prevalent in individuals of African descent compared to those of European ancestry. The risk of AF among blacks, has been independently associated with increasing percentage of European ancestry. In the Candidate-Gene Association Resource Study for every 10% increase in European ancestry, there was a 16% to 20% increased risk of AF. The prevalence of AF in Hispanic and Asians residing in the United States is also lower than in white individuals (103, 171–173).

Knowing that pancreatic transplantation is not a standard procedure and is only applicable for type 1 diabetes, addressing the disease duration is difficult. Therefore, despite the conflicting results noted above, we believe that optimal glycemic control is paramount to AF prevention. Table 6 provides more information from the studies noted above.

**Table 6:** AF and diabetes.

1st Author Ref. # Year	Cases/participants	Age, years	Follow-up, years (unless otherwise indicated)	Study type	Key findings/message
Gumptecht et al. (162) 2021	881/3,014	77.5 ± 8.06	Mean ECG monitoring interval 20.07 ± 8.98 days	Sample from the cross-sectional NOMED-AF study	• AF was identified in 22.6%. • AF prevalence greater in diabetics 25%; (95% CI, 22.5–27.8%) vs. non-diabetics 17%; (95% CI, 15.4–18.5%) respectively, *P* < 0.001. • Asymptomatic (silent) AF greater in diabetics 9%; (95% CI, 7.9–11.4 vs. non-diabetics 7%; 95% CI, 5.6–7.5, *P* < 0.001).
Huxley et al. (163) 2011	108,703/ 1,686,097	40–94	4.5–44	Systematic review and meta-analysis	• Patients with DM had an approximately 40% greater excess risk of AF compared to non-diabetic patients. • It was not possible to fully account for other known and putative risk factors. The authors suggested that the true risk between DM and subsequent risk of AF may be closer to 25%.
Dublin et al. (165) 2010	DM in 252/1,410 with AF DM in 311/2,203 Without AF	Median age for AF patients: 74 Median age for non-AF patients: 68	N/A	Population-based case-control study	• The risk of AF increased 3%/year in pharmacologically treated diabetics. Compared to non-diabetics, the adjusted odds ratios for AF in treated diabetics with an average hemoglobin A1c ≤7 was 1.06 (95% CI, 0.74–1.51); for A1c >7 but ≤8, 1.48 (95% CI, 1.09–2.01); for A1c >8 but ≤9, 1.46 (95% CI, 1.02–2.08); and for A1c >9, 1.96 (95% CI, 1.22–3.14). • OR for atrial fibrillation was 1.40 (95% CI, 1.15–1.71) for people with treated diabetes compared to those without diabetes. • The association between treated diabetes and atrial fibrillation was stronger in people who were obese (OR 1.64; 95% CI, 1.27–2.12)
Chang et al. (166) 2014	85,198/645,710	+metformin: 58.6 ± 17.1 −metformin: 57.0 ± 14.8	13	Data from Taiwan's Longtudinal cohort of diabetes patient data base 1999–2010	• The AF rate for the metformin user group was significantly lower than that of the non-user group (245 for metformin users and 293 for nonusers, *P* < 0.0001). • After adjustment the metformin group had a significantly lower AF occurrence rate with a HR of 0.81 (95% CI, 0.76–0.86, *P* < 0.001).
Chao et al. (167) 2012	4,137/12,065	+Thiazolidinediones (TZDs) 53.7 ± 12.0 −Thiazolidinediones (TZDs) 54.1 ± 12.2	63 ± 25 months	Data from Taiwan's National Health Insurance Research Database cohort	• The TZD group was associated with lower AF occurrence rate compared to the control group (1.2% versus 1.8%; *P*-value = 0.008). • After adjustment TZD could significantly lower the new AF occurrence rate (hazard ratio 0.69; 95% CI: = 0.49–0.91, *P* value = 0.028).
Saliba et al. (168) 2015	11,713/37,358	72 ± 14.5	1	Population-based historical cohort study	• Diabetic patients divided into 4 groups *via* Hgb A1c. • After adjusting for CHA_2_DS_2_-VASc score the two higher A1c groups were at greater risk of TIA or stroke (*P* < .001 for each group).
Ashburner et al. (169) 2016	Duration <3 years: 839/2,101 Duration >3 years: 1,261/2,101 A1c <7.0%: 883/1,933 A1c 7.0%–8.9%: 690/1,933 A1c >9.0%: 360/1,933	Duration <3 years: 69 ± 11 Duration ≥3 years: 71.7 ± 8.9 A1c <7.0%: 71.5 ± 9.6 A1c 7.0%–8.9%: 70.5 ± 9.7 A1c >9.0%: 67.9 ± 9.7	1	Data from the ATRIA cohort	• HgbA1c values were categorized at common clinical cut points: <7.0%, 7.0% to 8.9%, and ≥ 9.0%. • There was no significant increase in ischemic stroke rates using only baseline HgbA1c values. • After adjustment for stroke risk factors, there was an increased rate of ischemic stroke associated with ≥3 years diabetes duration (adjusted HR: 1.74, 95% CI, 1.10 to 2.76).
Overvad (170) 2015	17,018/137,222	Median age: 72.9	Median available follow-up time was 4.0 years.	Nationwide Danish Cohort Study	DM patients with the shortest duration (0–4 years) were at the lowest risk of thromboembolism. DM patients with the longest diabetes duration (≥15 years) appeared to be at the highest risk of thromboembolism (adjusted HR, 1.48; 95% CI, 1.29–1.70).

## Smoking

Use of tobacco has been associated with an increased risk of AF (103). Smoking has been has been identified as an AF risk factor across ethnicities and races (103, 174).

In the prospective, population-based Rotterdam Study, the association between smoking cigarettes and risk of AF development was examined in 5,668 subjects without baseline AF. During a median follow up of 7.2 years, AF was identified in 371 cases. After multivariate adjustment, current and former smokers had an increased relative risk (RR) of AF compared to subjects that never smoked (RR: 1.51, 95% CI, 1.07–2.12; and RR: 1.49, 95% CI, 1.14–1.97, respectively) (175).

In the Atherosclerosis Risk in Communities (ARIC) study, 15,329 participants were available for a smoking status analysis, and 15,078 were available for a cigarette-years and a combined smoking status and amount analyses. Subjects were classified as never, ever, and current smokers. AF incidence was 9.8% in ever smokers and 5.7% in never smokers. Multivariable-adjusted AF hazard ratios were (HR: 1.32; 95% CI, 1.10–1.57) in former smokers, (HR: 2.05; 95% CI, 1.71–2.47) in current smokers, and (HR: 1.58; 95% CI, 1.35–1.85) in ever smokers. Among participants with the highest amounts of cumulative smoking the risk of AF was 2.1 times higher than among those who never smoked (176).

The CHARGE-AF consortium collected and analyzed data from the Framingham Heart Study, ARIC, and the Cardiovascular Health Study [CHS]. AF incidence was 1.44 times higher in current smokers compared with nonsmokers (177).

The REasons for Geographic And Racial Differences in Stroke (REGARDS) Study examined the influence of secondhand exposure to tobacco on the risk of AF. A total of 2,503 participants (21%) reported environmental tobacco smoke exposure. Such exposure was significantly associated with AF (OR: 1.27; 95% CI, 1.08–1.50) (178).

A meta-analysis of 29 prospective studies suggested that smoking is associated with an increased risk of AF in a dose-dependent manner, but the association was stronger among current smokers compared to former smokers (179).

Although cigar and pipe smoking have been associated with cardiovascular disease, a link to AF has not been reported. In a 22-year follow-up study, both primary and secondary (former cigarette smokers) pipe/cigar smokers showed significantly greater risk of major coronary heart disease and stroke events compared to never smokers. There was little difference between the effects of primary and secondary pipe/cigar smoking after adjustment for differences in lifestyle and biological characteristics (180). A 2015 systematic review of the risks associated with cigar smoking linked it to increased all-cause and coronary heart disease mortality (181). Similarly, a 2018 study followed 357,420 participants who reported exclusively using cigar, pipes, or cigarettes or never using any type of tobacco product from 1985 to 2011. There was an elevated risk of death among exclusive current cigar and pipe users in relation to never tobacco users, although the hazard ratio for pipe users was statistically significant only in an age-adjusted model (182).

The use of alternative products, such as hookah, electronic cigarettes, vapes, e-hookahs, e-pipes and e-cigars is increasing. While use of hookah involves inhalation of flavored tobacco, the battery-operated electronic devices (heat-not-burn products) allow the user to breathe in nicotine through a vapor. The cardiovascular disease risk associated with these entities remains unclear (183, 184).

Smoking cessation counseling and support are recommended as an optimal approach to maintaining cardiovascular health and as an AF prevention strategy (103). Few smokers (∼4%) quit without assistance. A variety of nicotine replacement products are available to help. Table 7 below summarizes key studies linking AF to smoking.

**Table 7:** Cigarette smoking and AF.

1st Author Ref. # Year	Cases/participants	Age, years	Follow-up, years	Study type	Key findings/message
Staerk et al. (103) 2017	-----	-----	-----	Review	• In-depth review of AF epidemiology
Schnabel et al. (174) 2015	1544/9,611	Prevalence age adjusted	50	Cohort study	• During 50 years observed about a fourfold increase in the age-adjusted prevalence and more than a tripling in age-adjusted incidence of atrial fibrillation. • Frequency of smoking among individuals with new-onset AF has decreased
Heering et al. (175) 2008	371/5,668	Never smoked: 71.2 ± 9.8 Former smoker: 67.4 ± 7.6 Current smoker: 65.8 ± 7.4	7.2 (median)	Prospective, population-based	• Current and former smokers had increased risks of atrial fibrillation as compared to never smokers (RR: 1.51; 95% CI, 1.07–2.12; and RR: 1.49; 95% CI, 1.14–1.97, respectively). • In former smokers, increased smoking duration of was associated with AF risk in a way that was compatible with a dose-response relationship.
Chamberlain (176) 2011	876/15,329	Never smoked: 54.0 ± 5.8 Former smoker: 54.8 ± 5.8 Current smoker: 53.6 ± 5.7	13.1	Prospective investigation	• AF hazard ratios were: (HR:1.32; 95% CI, 1.10–1.57) in former smokers, (HR: 2.05; 95% CI, 1.71–2.47) in current smokers, and (HR:1.58; 95% CI, 1.35–1.85) in ever smokers. • In participants with the highest cumulative amounts of smoking the AF risk of was 2.1 times higher than among those who never smoked.
Alonso (177) 2013	Derivation cohorts: 1,186/18,556 Validation Cohorts: 585/7,652	60 ± 8 to 76 ± 6	5-year predictive model	Data from 5 cohorts	• A simple 5-year predictive model (including variables age, race, height, weight, systolic and diastolic blood pressure, current smoking, use of antihypertensive medication, diabetes, history of myocardial infarction, and heart failure) readily available in primary care settings adequately predicted AF in diverse populations.
O’Neal (178) 2015	2,503/12,021	65 ± 9.9	N/A	REGARDS Sub-study	• After adjustment for age, sex, race, income, and education, environmental tobacco smoke (ETS) exposure was associated with AF (OR = 1.30, 95% CI = 1.10–1.53)). After further adjustment for cardiovascular risk factors and potential confounders, the association between ETS exposure and AF remained statistically significant (OR = 1.27, 95% CI = 1.08–1.50). • A stronger association was observed between ETS exposure and AF for black (OR = 1.63, 95% CI = 1.27, 2.08) compared with white (OR = 1.03, 95% CI = 0.82, 1.30) participants (*P*-interaction = 0.0007).

## Alcohol

Alcohol consumption is strongly embedded in the food and societal culture of westernized countries (most countries of the European Union as well as the U.K., Norway, Iceland, Switzerland, the United States, Canada, Australia, and New Zealand) (185). Moderate to excessive alcohol drinking is associated with adverse atrial remodeling and incident AF. Adults who consumed 10 or more drinks containing approximately 12 g of pure alcohol weekly and who had paroxysmal or persistent AF on a rhythm control strategy were randomized in a 1:1 fashion to either abstain from alcohol or continue their usual alcohol consumption. In reality, the abstinence group reduced their intake by 87.5% and the continue their usual alcohol (control) group reduced their intake by 19.5%. The abstinence group had a significantly longer period before AF recurrence compared to the control group (*P* = 0.005) as well as a significantly reduced AF burden over 6 months follow up (*P* = 0.01) (186). Unfortunately, for many persons with AF, total abstinence from alcohol may be a difficult goal to achieve (185).

Binge drinking (defined as 5 or more drinks on at least one occasion for men or 4 or more drinks for women) is the most common and costly pattern of excessive alcohol use (187). In the United States (U.S.) in 2018, one in six U.S. adults reported binge drinking during the past 30 days (188). Binge drinking is on the rise among U.S. adults ≥65 years of age and in women (103). Interestingly, in a 2019 study that included a total of 9,776,956 Korean patients in its analysis, the number of drinking sessions per week was significantly associated with the developing new-onset AF, whereas there was a significant *inverse* relationship between the amount of alcohol consumed per drinking session and the risk of new-onset AF (189). Another Korean study, that included 9,797,409 subjects without a prior AF diagnosis, highlighted that heavy drinkers had a substantial risk of developing AF if aged ≥30 years old. Mild to moderate drinking increased the susceptibility to AF in subjects ≥60 years old (190, 191).

In the past, triggers of discrete AF episodes were poorly studied and incompletely characterized. In a study with 1295 participants designed to describe common AF triggers, 74% of these individuals reported triggers. Alcohol (35%) was the most commonly noted trigger (192). In order to further evaluate the hypothesis that acute alcohol consumption (drinking within a few hours before an episode) is independently associated with increased risk for a discrete AF episode, 100 patients aged ≥21 years with documented paroxysmal AF who consumed (on average) at least 1 standard alcoholic drink per month were equipped with wearable ECG monitors for 4 weeks. They were instructed to press an activator button on the ECG monitor only when and every time they had an alcoholic drink. In addition, they wore a transdermal alcohol sensor placed around the ankle for passive alcohol monitoring. On return clinic visits at 2 and 4 weeks a fingerstick blood spot was collected to test for phosphatidylethanol, an abnormal phospholipid formed in blood only in the presence of alcohol use. The Spearman correlation (statistical dependence between the rankings of two variables) between real-time recordings of alcohol consumption and daily areas under the curve for the transdermal alcohol sensor detected events was 0.52 (*P* < 0.001). Although no apparent threshold effects existed between the amount of alcohol consumed and risk for a discrete AF event, AF episodes were associated with increased blood alcohol concentration measured *via* the transdermal alcohol sensor during the previous 12 h (193).

Therefore, it is clear that alcohol consumption substantially increases the chance of a discrete AF episode within a few hours (193) and that alcohol is the most common precipitant of discrete AF episodes (194).

A summary of the studies related to alcohol is present in Table 8 below.

**Table 8:** Alcohol consumption and AF.

1^st^ Author Ref. # Year	Cases/participants	Age, years	Follow-up, years (unless otherwise indicated)	Study type	Key findings/messages
Voskoboinik et al. (189) 2020	70/140 Abstinence group: 70; Control group: 70.	62±9	6 months	Multicenter, prospective, open-label, randomized, controlled trial	• “Abstinence” group patients reduced their alcohol intake from 16.8±7.7 to 2.1±3.7 drinks/week. 61% achieved complete abstinence. • In the control group; alcohol intake was reduced from 16.4±6.9 to 13.2±6.5 drinks/week. At 6 months, AF had recurred in 37 abstinence patients (53%) and in 51 control patients (73%). Time to AF recurrence was longer in the abstinence group (HR: 0.55; 95% CI: 0.36 to 0.84; P=0.005). AF burden was significantly lower in the abstinence group (P=0.01).
Gillis (188) 2020	N/A	N/A	N/A	Editorial Comment on Voskoboinik et al. (above)	• Moderate-to-heavy alcohol (ETOH) consumption is strongly associated with incident atrial fibrillation in both men and women. Alcohol has been reported to be a common trigger for PAF. • Raises important questions: Abstinence is effective for PAF, but is it sustainable? Is abstinence effective if the AF burden is high? Would moderation work?
Kim et al. (192) 2020	195,829/9,776,956 developed AF	No ETOH: 50.65±14.28 Mild ETOH: 42.89 ± 12.89 Moderate ETOH: 43.52 ± 12.32 Heavy ETOH: 44.29 ± 12.68	2009 checkup date until to the date of diagnosis of new-onset AF or December 2017 if no new-onset AF was detected.	Population-based study from the [South] Korean National Health Insurance Service (K-NHIS) database	• Influence of drinking frequency (day per week), alcohol consumption per drinking session (grams per session), and alcohol consumption per week were studied. • Heavy drinkers had the greatest risk (21.5%) for new-onset AF (HR 1.215, 95% CI 1.193–1.238). The number of drinking sessions/week was associated with the risk of new-onset AF. • The amount of alcohol consumed/drinking session was inversely related to new-onset AF. • Binge drinking was not associated with new-onset AF.
Groh et al. (195) 2019	957/1295 of patients with symptomatic paroxysmal AF reported triggers	63	Via online survey	Report of respondents recruited from patients enrolled in the Health eHeart Study and through the patient-centered advocacy organization StopAfib.org	• The most common reported AF triggers were alcohol (35%), caffeine (28%), exercise (23%), and lack of sleep (21%). • Younger patients, women, and those with an AF family history more commonly experienced various triggers. • Female sex, Hispanic ethnicity, obstructive sleep apnea each were associated with a greater number of AF triggers.
Marcus et al. (196) 2021	56/100	64 ± 15	4 weeks	Prospective, case-crossover analysis	• Participants wore a continuous electrocardiogram (ECG) monitor and had an ankle-worn transdermal ethanol sensor for 4 weeks. Real-time documentation of each alcoholic drink was self-recorded via a button on the ECG monitor. • Fingerstick blood tests for phosphatidylethanol (PEth) were used to corroborate drinking events. • An AF episode was associated with 2-fold higher odds of 1 alcoholic drink (OR: 2.02; 95% CI: 1.38 to 3.17]) and > 3-fold higher odds of at least 2 drinks (OR: 3.58; 95% CI: 1.63 to 7.89) in the preceding 4 hours.
Aung et al. (197) 2022	1,269,054 breath alcohol measurements were obtained from 36,158 individuals	N/A	N/A	Instrumental variable analysis	• Analyzed breathalyzer data and identified eight recurrent and nationally recognized events associated with increased alcohol consumption. • Compared rates of AF and new-onset AF during and 6 d after instrumental variable events compared with all other days of the year, alcohol consumption inferred from these events was associated with a statistically significant increase in ED visits for AF and for new-onset AF. • The data suggests that acute alcohol consumption in the general population is associated with a higher risk of discrete AF episodes as well as for new-onset AF.

## AF and sleep disordered breathing

It has been estimated that the global prevalence of obstructive sleep apnea approaches a billion individuals (195). Obstructive sleep apnea (OSA) or obstructive sleep apnea-hypopnea syndrome (OSAHS) (196) is highly prevalent among AF patients. In population-based studies, the prevalence of OSA ranges from 3% to 49% and from 21% to 74% in AF patients (197). It remains uncertain whether AF is a risk factor for OSA (198).

A meta-analysis of 8 studies including 603,532 non-OSAHS and 14,799 OSAHS patients revealed that OSAHS increased the risk of AF (RR = 1.70, 95% CI, 1.53–1.89, *P* = 0.002). There was a significant association between mild SAHS and the risk of AF (RR = 1.52, 95% CI, 1.28–1.79, *P* = 0.01), moderate SAHS (RR = 1.88: 95% CI, 1.55–2.27, *P* = 0.017), and severe SAHS (RR = 2.16, 95% CI, 1.78–2.62, *P* < 0.001). These results suggested that the greater the OSAHS severity, the higher the risk of AF (199). Unfortunately, a meta-regression analysis to evaluate the influence of variables such as a history of cardiovascular disease and body mass index on AF risk was not performed because these variables were unavailable in the studies included (196, 199).

Youssef and associates performed a meta-analysis of nine observational studies with a pooled sample size of 7,582 non-OSAHS and 12,255 OSAHS patients. The risk of AF was higher in OSAHS group vs. the control group (OR: 2.1, 95% CI, 1.84–2.43, *P*: < 0.001) (200). The meta-analysis had several limitations. The study design of ∼40% of studies was cross-sectional which could limit the ability to make conclusions about the impact of OSAHS on AF incidence. In addition, quality scores for the included studies were not reported and other confounding factors were not examined using meta-regression analysis (196, 200).

It is unclear whether treatment of sleep disorders has an impact on AF incidence (196). However, benefits from continuous positive airway pressure (CPAP) therapy post-AF catheter ablation have been noted. Patients who receive CPAP therapy appear to have a lower risk of AF recurrence after AF ablation and cardioversion (103, 196, 197, 201).

In a multi-center study 3,000 patients underwent AF catheter ablation. OSA was present in 640 (21.3%). Overall, the OSA group was noted to have more non-pulmonary vein AF triggers (HR = 1.68, 95% CI, 1.12–2.52, *P* < 0.009).

During a mean follow up of 32 ± 14 months, 78% of the non-OSA group were free of AF vs. 73% in the OSA group (*P* = 0.024). Among the OSA patients, the non-CPAP group had more early recurrences than the CPAP group: 178 (55%) vs. 105 (33%) respectively (*P* < 0.001). Paroxysmal AF patients who used CPAP had 31 (20%) procedural failures compared with 36 (33%) of the non-CPAP group (*P* = 0.019). Non-paroxysmal AF patients who used CPAP had 128 (79%) success vs. 150 (68%) in a non-CPAP population (*P* = 0.032) (202).

Fein and associates reported the results from 426 (62 with OSA) patients who underwent pulmonary vein isolation procedures for AF between 2007 and 2010 and were followed for 1 year. Thirty-two of the 62 (51.6%) with OSA used CPAP and 30 (48.4%) did not. The AF recurrence rate in CPAP users was similar to patients without OSA. In non-users AF recurrence was significantly higher (HR: 2.4, *P* < 0.02) and similar to that of OSA patients managed without ablation (HR: 2.1, *P* = 0.68) (203).

During a follow-up period of 18.8 ± 10.3 months, Naruse et al. also reported higher AF recurrence rates post-ablation among 34 untreated OSA patients compared to 82 CPAP users. They concluded that appropriate treatment with CPAP in patients with OSA is associated with reduced recurrence of AF (204).

In a 2014 meta-analysis of 5 observational studies (including 3743 patients), individuals with OSA had a 31% greater risk of AF recurrence after catheter ablation compared to those without OSA [relative ratio (RR) = 1.31, *P* = 0.00]. This risk increased by 57% in patients with OSA not receiving CPAP therapy (RR = 1.57, *P* = 0.00). CPAP users had a risk of AF recurrence similar to that of patients without OSA (RR = 1.25, *P* = 0.37). This similarity was maintained after the removal of study heterogeneity (205).

It has been noted that most of the data gathered on AF and sleep disordered breathing has been observational. In contrast, Hunt et al. randomized patients with paroxysmal AF and an apnea-hypopnea index >15 events/hour who underwent pulmonary vein isolation to treatment with CPAP (in 37patients) or standard care (in 46 patients). Treatment with CPAP did not further reduce the risk of recurrent AF after ablation (206). Nevertheless, the preponderance of evidence suggests a concordant relationship between the severity of sleep disordered breathing and AF incidence, burden, and therapeutic response. Individuals with severe sleep disordered breathing are less likely to respond to antiarrhythmic drug therapy than those with milder forms (207).

Although underpowered for AF, the large multicenter randomized SAVE study (Sleep Apnea Cardiovascular Endpoints), that compared CPAP to usual care alone and did not show a reduction in cardiovascular events, including incident new-onset AF, in patients with moderate to severe OSA and established cardiovascular disease. Nevertheless, until more randomized data is available, screening for concomitant sleep disordered breathing should be considered important in AF patients because treatment may decrease their AF burden. Clinicians need to be aware that, in sleep apnea, patient compliance with CPAP can be difficult to achieve (208).

While the overall benefit of CPAP on AF is not significant, the use of CPAP may reduce the risk of AF recurrence post ablation. A collection of studies that pertain to OSA and sleep apnea is summarized in Table 9 below.

**Table 9:** AF and sleep disordered breathng.

1^st^ Author Ref. # Year	Cases/participants	Age, years	Follow-up, years (unless otherwise indicated)	Study type	Key findings/messages
Zhao et al. (199) 2018	14,799/618,331	Minimum mean age 38.9 and maximum mean age 75.0.	2 years 4 months to a mean of 9.2 years	Meta-analysis	• Sleep apnea increased the risk of AF significantly. • After adjustment for confounders, the relative risk (RR) of AF was 1.40 (95% CI: 1.12–1.74, *P* < 0.001)
Yousef et al. (200) 2018	1404/19,837 (entire group) + OSA: 943/12,255 - OSA:461/7582	N/A	N/A	Meta-analysis	• The risk of AF was found to be higher among OSA versus control group (OR; 2.120; CI: 1.845-2.436, P<0.001).
Monahan et al. (207) 2012	61/61 (atrial fibrillation and flutter)	64 ± 9	1	Cohort study	• Anti-arrhythmic drug (AAD) response defined as: remaining on the same AAD for ≥ 6 months with ≥75% reduction in symptomatic AF burden. • 49% were responders. The response rate was twice as high in those with non-severe OSA compared to those with severe OSA (61 vs 30%; p = 0.02).
McAvoy et al. (208) 2016	+CPAP:1359/2717 -CPAP: 1358/2717	61	Mean 4.5	Secondary prevention trial	• Mean duration of CPAP adherence dropped from 4.4±2.2 hours per night to 3.5±2.4 hours at 1 year. • In this large group of adults with both cardiovascular disease and moderate-to-severe obstructive sleep apnea, the use of CPAP therapy had no significant effect on the prevention of recurrent serious cardiovascular events
Patel et al. (202) 2010	640/3000 +CPAP 315 -CPAP 325	+OSA 51 ± 10 -OSA 57 ± 11	32±14 months	Multicenter cohort study	• Patients with OSA had a higher prevalence of non-PV triggers (20% versus 8%, *P*<0.001). Non-paroxysmal AF patients without OSA had fewer non-PV triggers compared with patients with OSA (19% versus 31%, *P*<0.001). • Success was achieved in 78% of the non-OSA group compared with 73% in the OSA group (*P*=0.024)
Fein et al. (203) 2013	Ablation group: (PVI) 62/426 +CPAP 32 -CPAP 30 Control group: -CPAP 30 Medical Rx (No PVI) +CPAP	Ablation +CPAP: 56.8 ± 1.2 Ablation -CPAP: 58.5 ± 1.4 Med Rx +CPAP 56.0 ± 1.8	1	Prospective cohort study	• Post ablation, patients were treated with AADs (usually class IC agents or sotalol for 3-6 months. • Post ablation atrial tachyarrhythmia–free survival rate was significantly higher in the +CPAP group compared with the -CPAP Group (71.9% vs. 36.7%; P = 0.01. • Arrhythmia-free survival without AADs/repeat ablation was significantly higher in the +CPAP group compared with the -CPAP group (65.6% vs. 33.3%; P = 0.02).
Naruse et al. (204) 2013	153/209 116 +OSA 37 -OSA 82/116 +CPAP 34/116 -CPAP	60 ± 9	Mean 18.8 ± 0.3 months	Prospective cohort study	• 51 (33%) patients experienced AF recurrences after ablation. • Recurrence: +OSA (hazard ratio 2.61; 95% CI: 1.12–6.09; P < .05). • Appropriate CPAP treatment in OSA patients is associated with a lower recurrence of AF (HR 0.41; 95% CI: 0.22–0.76; P < .01)
Li et al. (205) 2014	3473	51.0 ± 10 to 60 ± 8	7-20 months	Meta-analysis	• OSA patients had a 31% greater risk of AF recurrence after successful catheter ablation than the non-OSA patients. • Risk of recurrent AF increased by 57% in patients who did not receive CPAP therapy. • OSA patients who received CPAP therapy had a risk of recurrent AF similar to that of the non-OSA patients.
Hunt et al. (206) 2022	83 total +CPAP 36 - CPAP 47	Total 62 ± 8 +CPAP 62 ± 8 -CPAP 62 ± 7	1 year	Randomized Trial	• PVI reduced AF burden in OSA patients. • CPAP did not improve risk of AF recurrence after ablation beyond the benefit of PVI.

## Caffeine

Despite an absence of supportive evidence, many clinicians continue to recommend that patients with atrial arrhythmias avoid coffee and other caffeinated beverages (209). Table 10 summarizes 13 studies examining the relationship between caffeinated beverages and atrial arrhythmia (209–222). A small case-control study without adjustments for confounders reported that coffee was detrimental (211). The remaining studies (210, 212–222) showed benefit or no significant interaction (209).

**Table 10:** Caffeine and atrial arrhythmias.

1st Author Ref. # Year	Cases/participants	% Males	Age (years)	Follow-up (years)	Study Design	Key findings
Wilhelmsen et al. (210) 2001	754/7,495	100	47 ± 55	25.2	Population-based cohort study	• Coffee consumption was not associated with an increased risk of hospitalization for AF. • Compared to nondrinkers, (OR: 1.24; 95% CI, 1.00 to 1.54) for 1–4 cups/day, and OR: 1.09; 95% CI, 0.87 to 1.38) for ≥5 cups/day.
Mattioli (211) et al. 2005	116/232	74	54 ± 7	–	Case control	• Higher coffee intake, >3 cups/day, was associated with a lower rate of spontaneous conversion from AF (OR: 0.3; 95% CI, 0.11 to 0.49; *P* = 0.008).
Frost et al. (212) 2006	555/47,949	47	56	5.7	Prospective cohort	• Compared with the lowest quintile, there was no association between caffeine intake and incident AF (lowest risk was third quintile, ∼584 mg/day; HR: 0.85; 95% CI, 0.65 to 1.12).
Mukamal et al. (213) 2009	163/1,369	70	59.8	6.9–9.9	Prospective cohort	• In patients with previous myocardial infarction, coffee drinkers in the 4 highest categories of intake had ∼30% lower risk of developing AF (HR for ≥1 cup/day: 0.65; 95% CI, 0.40 to 1.05).
Conen et al. (214) 2010	945/33,638	0	53	14.4	Prospective cohort	• U-shaped relationship with lowest risk of incident AF in third quartile of intake with median 285 mg/day (HR: 0.78; 95% CI, 0.64 to 0.95; *P* for quadratic trend = 0.03). • None of the individual caffeine components (tea, coffee, cola, chocolate) was associated with incident AF.
Shen et al. (215) 2011	296/4,526	44	62 ± 10	4.0	Prospective cohort	• No correlation between caffeine intake and risk of incident AF for quartiles of intake (for Q1 HR: 0.84; 95% CI, 0.62 to 1.15 vs. Q4 HR: 0.98; 95% CI, 0.7 to 1.39; *P* for trend = 0.84).
Klatsky et al. (216) 2011	1,512/130,054	44	–	17.6	Retrospective population cohort	• Higher coffee intake was associated with lower rates of hospitalization for AF (HR: 0.81; 95% CI, 0.69 to 0.96 for ≥4 cups/day) and SVT (HR: 0.63; 95% CI, 0.41 to 0.98 for ≥4 cups/day).
Caldeira et al (217) 2013	Total of 115,993 participants	–	51–62	4–25	Meta-analysis	• There was no significant association between caffeine exposure and AF risk (OR 0.92; 95% CI, 0.82 to 1.04). • People with low caffeine intake may be at lower risk of AF (OR 0.85; 95% CI, 0.78 to 0.92). • After excluding “poorer quality” studies the pooled estimate became significant with a 13% reduction in the odds of AF among caffeine consumers (OR 0.87; 95% CI, 0.80 to 0.94).
Cheng et al. (218) 2014	4,261/228,465	46	53–63	4–25.2	Meta-analysis	• The pooled RR for AF incidence of habitual caffeine exposure was 0.90 (95% CI, 0.81–1.01; *P* = 0.07). • In subgroup analyses based on the level of caffeine intake, low and high doses of caffeine intake reduced AF risk by 6% (RR, 0.94; 95% CI, 0.83–1.07; *P* = 0.02) and 12% 12% (RR, 0.88; 95% CI, 0.78–0.99, *P* = 0.13). • In subgroup analyses based on comparability bias, habitual caffeine intake reduced AF incidence by 14% (RR, 0.86; 95% CI, 0.78–0.94, *P* = 0.002)
Larsson et al. (219) 2015	7,041/76,475	55	61.5	12	Population-based cohort study	• No association between coffee consumption and risk of incident AF at all levels of consumption (multivariate RR: 0.98 for 2–3 cups/day; RR: 1.01 for ≥5 cups/day; *P* = 0.64 for trend).
Liu et al. (220) 2016	401/801	56	63 ± 12	–	Case control	• Green tea was protective against incident AF (multivariate OR: 0.349; 95% CI, 0.25 to 0.48) in a dose-dependent manner (*P* for trend = <0.001).
Dixit et al. (221) 2016	1,388	47	71.9 ± 5	N/A	Observational study (24-hr Holter)	• No correlation between atrial ectopics/h and intake of coffee (*P* = 0.28) or tea (*P* = 0.57). • No correlation between SVT runs and intake of coffee (*P* = 0.22) or tea (*P* = 0.90).
Mostofsky et al. (222) 2016	3,415/57,053	48	56.7 ± 4.4	13.5	Population-based cohort study	• Higher coffee intake was associated with lower rate of incident AF (linear trend: *P* = 0.02). • Compared to nondrinkers, (OR: 0.86; 95% CI, 0.71 to 1.04) for 2–3 cusps per day, and (OR: 0.79; 95% CI, 0.64 to 0.98) for 6–7 cups per day.

## AF in ischemic and structural heart disease

The prevalence of AF among patients with CAD has been estimated to range from 0.2% to 5%. In contrast, the reported prevalence of CAD in patients with AF has ranged from 17% to 46.5% (223). In a meta-analysis of 43 myocardial infarction (MI) studies (including 278,854 subjects) which evaluated mortality related to AF, both new onset and preexisting AF conferred an increased likelihood of death. The reported incidence of new AF was 10% and the incidence of prior AF was 7%. A similar significant association between AF and mortality was noted when the analysis was performed for new AF and prior AF individually. The mortality OR for new AF was 1.37 (95% CI, 1.26 to 1.49) and for prior AF was 1.28 (95% CI, 1.16 to 1.40). The follow up time varied widely across studies but was primarily ≤ the duration of patients' hospital stay (224).

A smaller study of 3,220 patients hospitalized with MI revealed prior AF in 304 (9.4%) and new AF in 729 (22.6%). New AF post MI occurred in 218 patients [30%] within 2 days, 119 [16%] between 3 and 30 days, and 392 [54%] after >30 days. During a mean follow-up of 6.6 years, AF was associated with an increased risk of mortality (hazard ratio, 3.77; 95% confidence interval 3.37 to 4.21) compared to patients without AF. The risk of death was highest when AF occurred >30 days post MI (hazard ratio, 2.58; 95% confidence interval 2.21 to 3.00) (225).

In a 2003 report, The Global Registry of Acute Coronary Events (GRACE) examined the relationship between AF and acute coronary syndromes (ACS), including ST segment elevation acute MI, non-ST segment elevation acute MI, and unstable angina (defined as presence of new or accelerated ischemic symptoms with or without electrocardiographic changes, but without elevation of cardiac enzymes). AF included both prior and new-onset atrial fibrillation and atrial flutter.

Compared with patients without AF, those with new-onset AF were more likely to have had an anterior or any ST-segment elevation MI and cardiac arrest on hospital arrival. While patients with ACS and any AF had worse in-hospital outcomes than those without any AF, all complication rates were higher in patients with ACS and new-onset AF than in those with prior AF. Patients with new AF were significantly more likely to receive temporary pacing, mechanical ventilation, a pulmonary artery catheter, and an intra-aortic balloon pump. Cardiac catheterization rates were similar, coronary artery bypass surgery was more frequent and percutaneous intervention (PCI) less frequent. Among patients with prior AF, cardiac catheterization, PCI, and coronary artery bypass surgery were employed less frequently. Use of temporary pacing, pulmonary artery catheterization, and intra-aortic balloon pumps were statistically less frequent, but percentagewise quite similar to non-AF patients. All AF patients were more likely to experience pulmonary edema, cardiac arrest and death. New AF patients were also more likely to suffer a stroke, reinfarction, major bleeding, and have a longer length of hospital stay (226).

More recently, it has been estimated that 5%–15% of AF patients will require PCI/stenting at some point in their lives. In these individuals, it is pivotal that the risk of bleeding be balanced against the risk of stent occlusion/reinfarction/ACS and/or cerebral infarction. It has been suggested that triple therapy (DAPT + an oral anticoagulant) may be continued for up to 6 months post ACS if the HAS-BLED score is <3. Dual therapy with an a single antiplatelet + oral anticoagulant (a P2Y12 inhibitor is favored over aspirin) should be used over the next 6 months and at 1 year followed by lifelong use of an oral anticoagulant. If the HAS-BLED score is ≥3 a duration of 1 month of triple therapy has been recommended. Dual therapy with an a single antiplatelet + oral anticoagulant is recommended for the next 11 months and at 1 year followed by lifelong use of an oral anticoagulant.

After elective PCI, triple therapy is appropriate for 1 month regardless of the HAS-BLED score. If the HAS-BLED score is <3, dual therapy with an a single antiplatelet + oral anticoagulant (a P2Y12 inhibitor is favored over aspirin) is recommended for the next 11 months and at 1 year is followed by lifelong use of an oral anticoagulant. If the HAS-BLED score is ≥3, following 1 month of triple therapy with 5 months of dual therapy is reasonable followed by lifelong use of an oral anticoagulant (223).

AF with a rapid ventricular response may be accompanied by ST segment depression and symptoms suggesting ischemia in the absence of significant obstructive coronary artery disease. Even troponin release may occur in the absence of obstructive CAD. AF has also been independently linked with up to a 3-fold increased risk of cardiac arrest due to VF. The mechanism is likely multifactorial and may result from one or more of the following: a direct proarrhythmic effect of AF, an increased cardiac workload, tachycardia-induced ischemia, or heart failure (227).

Structural heart disease typically refers to non-coronary cardiovascular disease and related interventions. Screening for structural heart disease has an important role in the care of AF patients.

Steinberg and associates have described a spectrum of structural heart disease which including valvular heart disease and paravalvular leaks, hypertrophic cardiomyopathy, atrial (including patent foramen ovale) and ventricular septal defects, patent ductus arteriosus, the left atrial appendage (as a source of thromboemboli) and left ventricular aneurysm (which is usually related to myocardial infarction) (228). A detailed discussion of each of these entities is beyond the scope of this discussion. Not all experts agree with the spectrum as described.

The consequences of structural heart disease may lead to heart failure (HF). The risk of AF increases 4.5 to 5.9-fold in the presence of HF and AF is present in more than 15% of HF patients. The prevalence of AF increases as HF severity worsens (5%–10% in mild HF, 10%–26% in moderate HF, and up to 50% in advanced HF). As many as 25%–35% of individuals with decompensated HF present in AF. Among patients with HF, AF develops at a rate of 6%–8%/year. AF may precipitate HF exacerbation and HF may trigger AF (229).

The key features of AF management in HF include optimizing HF medical management, assessing thromboembolic risk and anticoagulation, rate control (pharmacological or *via* AV junction ablation) and evaluating the need for cardioversion and maintenance of sinus rhythm as well as choosing the method to achieve it (pharmacological, catheter or surgical ablation). Recently, greater emphasis has been placed on using rhythm control (typically with antiarrhythmic drugs and/or AF ablation) rather than rate control to reduce adverse cardiovascular outcomes. Evidence increasingly supports early rhythm control for AF that has not become long-standing. Early rhythm control may reduce irreversible atrial remodeling and prevent AF-related deaths, heart failure, and strokes in high-risk patients. In patients with HF and AF, catheter ablation may be preferred to AADs due to challenges in optimizing pharmacological strategy in this population (40, 229, 230).

Cardiomyopathic disorders are not due to coronary disease, hypertension, and congenital, valvular, or pericardial abnormalities. Cardiomyopathies may be divided into 4 main subtypes hypertrophic, dilated, restrictive, and arrhythmogenic right ventricular cardiomyopathy. In a retrospective cohort study of 634,885 cardiomyopathy patients, concomitant AF was present in 14,675 (2.3%) patients with hypertrophic, 90,117 (7.0%) with restrictive, and 37,685 (5.9%) with dilated cardiomyopathy. Significantly higher odds of hospitalization, incident HF, and stroke were noted in all cardiomyopathy subtypes. AF was associated with significantly greater odds of all-cause mortality in hypertrophic (OR:1.26; 95% CI, 1.13–1.40) and dilated [1.36 (1.27–1.46)], but not restrictive [0.98 (0.94–1.02)], cardiomyopathy. AF catheter ablation was associated with significantly lower odds of all-cause mortality at 12 months across each of the cardiomyopathy subtypes (231).

**Figure 4:**
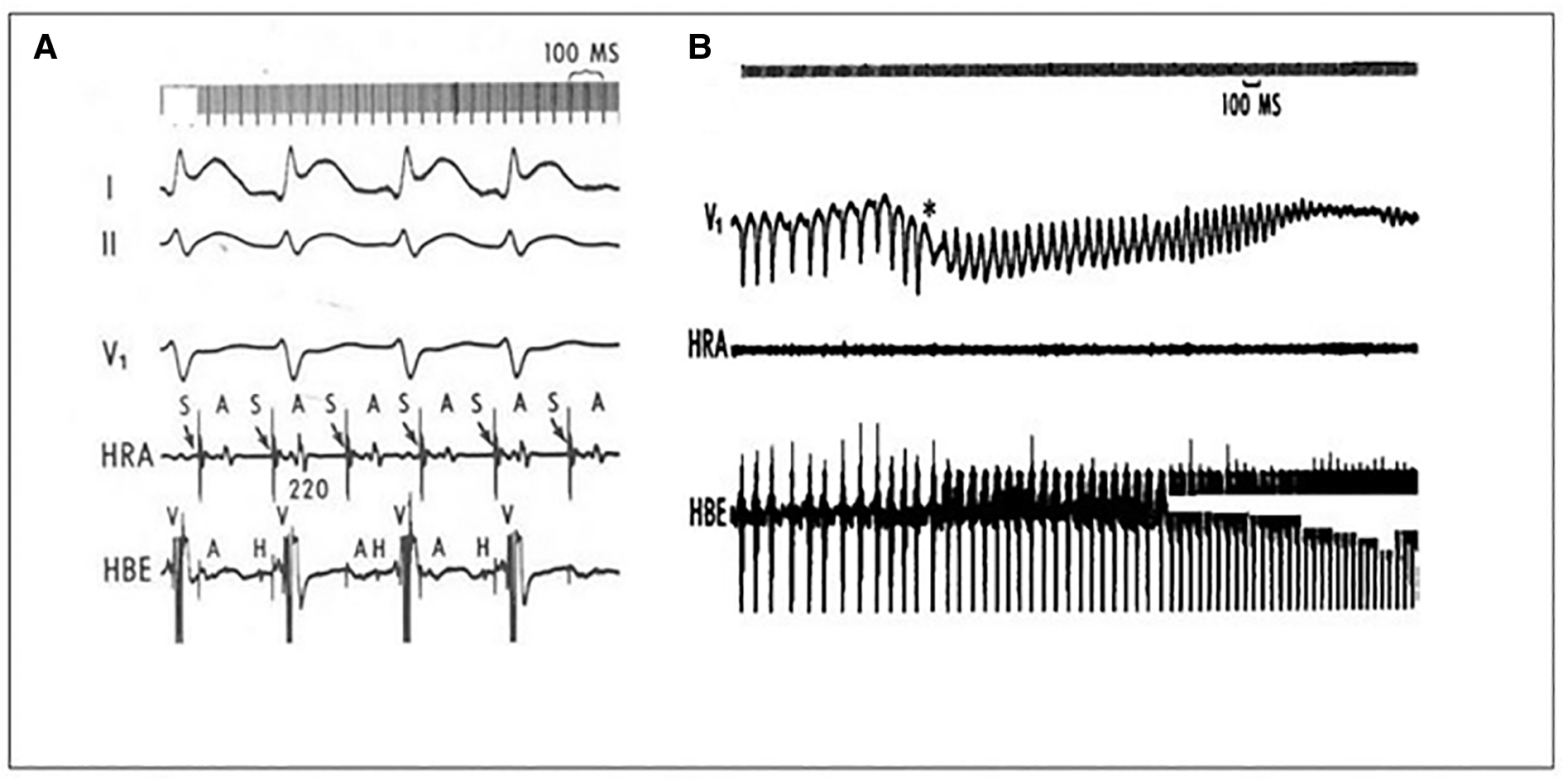
(**A**) during rapid pacing ST segment elevation is present. (**B**) Atrial fibrillation with a rapid ventricular response was induced. 100 s later, the rhythm degenerated into ventricular which was terminated with a 360 joule shock. Adapted from reference (59) with permission.

The most dramatic example we have seen was of AF's adverse influence on cardiomyopathy was reported in 1986. A 15-year-old male with non-obstructive hypertrophic cardiomyopathy suffered an out of hospital cardiac arrest. He was evaluated with invasive electrophysiologic testing. Although, no ventricular arrhythmias were inducible, we had observed that transient ST elevation occurred during rapid atrial pacing (Figure 4A). Near the procedure's end, rapid right atrial pacing at progressively accelerating rates induced AF with a ventricular rate of 180–190 beats/min. The patient complained of substernal constricting chest discomfort. After 100 seconds of this arrhythmia, there was a sudden degeneration of the rhythm to ventricular fibrillation (VF) requiring electrical defibrillation with a 360 Joule shock to restore sinus rhythm (Figure 4B).

He was discharged on a combination of metoprolol and verapamil therapy. During a follow-up period of 5 months, he had one asymptomatic spontaneous AF episode. The ventricular rate was 95 to 100/min, and he was hemodynamically stable. Direct current cardioversion restored sinus rhythm. To the best of our knowledge, this was the first report of AF progressing to VF in the absence of preexcitation (59).

Table 11 summarizes key points about the relationships between ischemic and structural heart disease and AF.

**Table 11:** Ischemic, structural heart disease and AF.

1st Author Ref. # Year	Cases/participants	Age, years	Follow-up, years (unless otherwise indicated)	Study type	Key findings/message
Michniewicz et al. (223) 2018	N/A	N/A	N/A	Review	• Prevalence of AF among patients with CAD has been estimated to range from 0.2% to 5%. • Reported prevalence of CAD in patients with AF has ranged from 17% to 46.5%. • Patients with ACS and AF have had worse in-hospital outcomes than those without any AF. Complications are higher with new onset vs. prior AF.
Jabre et al. (224) 2011	278,854 with AF and MI	N/A	Varied widely among studies	Systematic review and meta-analysis	• There was a significant association between AF and mortality (OR: 1.46; 95% CI, 1.35–1.58). • Mortality for new AF (OR: 1.37; 95% CI, 1.26–1.49). Mortality for prior AF (OR: 1.28; (5% CI, 1.16–1.40).
Mehta et al. (226) 2003	2921/21,785	N/A	In-hospital	Sample from Global Registry of Acute Coronary Events (GRACE)	• Patients with ACS and any AF were more likely to have a complicated in-hospital course than those without AF. • With new-onset AF there was a 2.5- to 4-fold increase in the adverse events of reinfarction, cardiogenic shock, pulmonary edema, cardiac arrest, major bleeding, and stroke. • As a result, in-hospital death rates were increased nearly threefold in patients with new-onset AF. Importantly, almost all complications remained higher in patients with new-onset AF compared with no AF. • In contrast, although in-hospital mortality was increased twofold in patients with previous AF the independent effect of previous AF on in-hospital mortality was not upheld when potentially confounding prognostic factors were controlled for in a multivariate regression analysis.
Šmíd et al. (227) 2017	N/A	N/A	N/A	Review	• AF with a rapid ventricular response may be accompanied by ST segment depression and symptoms suggesting ischemia in the absence of significant obstructive coronary artery disease. • Even troponin release may occur in the absence of obstructive CAD. • AF has been independently linked with up to a 3-fold increased risk of cardiac arrest due to VF. • The mechanism for VF may result from one or more of the following: a direct proarrhythmic effect of AF, an increased cardiac workload, tachycardia-induced ischemia, or heart failure. • Mortality is 2–4-fold higher in individuals with AF.
Darby et al. (229) 2012	N/A	N/A	N/A	Review	• The risk of AF increases 4.5 to 5.9-fold in the presence of heart failure (HF) and AF is present in >15% of HF patients. • The prevalence of AF increases as HF severity worsens (5–10% in mild HF, 10–26% in moderate HF, and up to 50% in advanced HF). • 25%–35% of individuals with decompensated HF present in AF. • Among patients with HF, AF develops at a rate of 6–8%/year. • AF may precipitate HF exacerbation and HF may trigger AF. • AF management in HF includes optimizing HF medical management, assessing thromboembolic risk and anticoagulation, rate control and evaluating the need for cardioversion and maintenance of sinus rhythm as well as choosing the method to achieve it.
Buckley et al. (231) 2021	142,477 /634,885	HCM +AF: 52.6 ± 20.0 HCM -AF: 65.3 ± 14.6 DCM +AF: 66.5 (13.7) DCM -AF: 57.0 ± 17.5	At least 1	Retrospective cohort study	• Concomitant AF was present in 14,675 (2.3%) patients with hypertrophic, 90,117 (7.0%) with restrictive, and 37,685 (5.9%) with dilated cardiomyopathy. • Significantly higher odds of hospitalization, incident HF, and stroke were noted in all cardiomyopathy subtypes. • AF was associated with significantly greater odds of all-cause mortality in hypertrophic (OR:1.26; 95% CI, 1.13–1.40) and dilated [1.36 (1.27–1.46)], but not restrictive [0.98 (0.94–1.02)], cardiomyopathy. • AF catheter ablation was associated with significantly lower odds of all-cause mortality at 12 months across each of the cardiomyopathy subtypes.

## Multiple morbidities and prevention of sequelae

Some individuals with AF, particularly older people, are asymptomatic. Many experience one or more of the following: palpitations (irregular beats, rapid rates), dyspnea, reduced exercise tolerance, lightheadedness, and chest pain. AF is associated with an increased risk of all-cause mortality, stroke, higher medical costs and a reduced quality of life (232, 233). Odutayo and associates performed a meta-analysis of 104 eligible cohort studies involving 9,686,513 participants (AF in 589,867 subjects). AF was associated with increased all-cause mortality, cardiovascular mortality, major cardiovascular events, any stroke, ischemic stroke, hemorrhagic stroke, ischemic heart disease, sudden cardiac death, congestive heart failure, chronic kidney disease, and peripheral arterial disease. Among these, the pooled relative risk of incident congestive heart failure was highest (RR:4.99; 95% CI, 3.04–8.22) (234).

Risk factors often do not occur in isolation. In the (previously noted) mAFA-II Randomized Clinical Trial, 833 AF patients with multiple morbidities used a mobile health (mHealth) technology that implemented the ABC pathway and 1,057 AF patients with multiple morbidities were allocated to usual care. The composite outcome of stroke or thromboembolism, all-cause death, and rehospitalization was significantly reduced in the ABC intervention group (*P* < .001). Likewise, sole analysis of rehospitalization also revealed significant reduction (*P* < .001). Analysis of the C component of the pathway (Comorbidities and Risk Factors) demonstrated that, during follow-up, rates of uncontrolled blood pressure, heart failure and acute coronary syndrome were lower in the intervention group (*P* < .001). Subgroup analyses by age, prior stroke, and sex demonstrated consistently lower hazard ratios for the primary composite outcome and rehospitalization in the intervention patient group (233).

## The way various risk factors impact AF incidence and progression

Although structural remodeling is a common end point of most AF-promoting risk factors, the process occurs slowly. Slow progressive structural remodeling due to advancing age and comorbidities such as hypertension likely contribute strongly to AF maintenance and progression (i.e., from paroxysmal to persistent or permanent AF) (235).

Nevertheless, it is important to understand that fluctuations in triggers and/or components of the substrate that occur transiently can result in temporal variability in AF risk. Although speculative, it seems likely that partial/incomplete recovery may from transient disturbances may ultimately lead to an accumulation of progressive AF risk (235).

It has become clear that some risk factors exhibit strong temporal variability. Examples of transiently increased AF risk include heart failure exacerbation and inflammation post-cardiac surgery. Several other AF risk factors may demonstrate a high day-to-day variability (e.g., sleep apnea) or may occur only during specific conditions such as exercise-induced hypertension (235).

**Figure 5:**
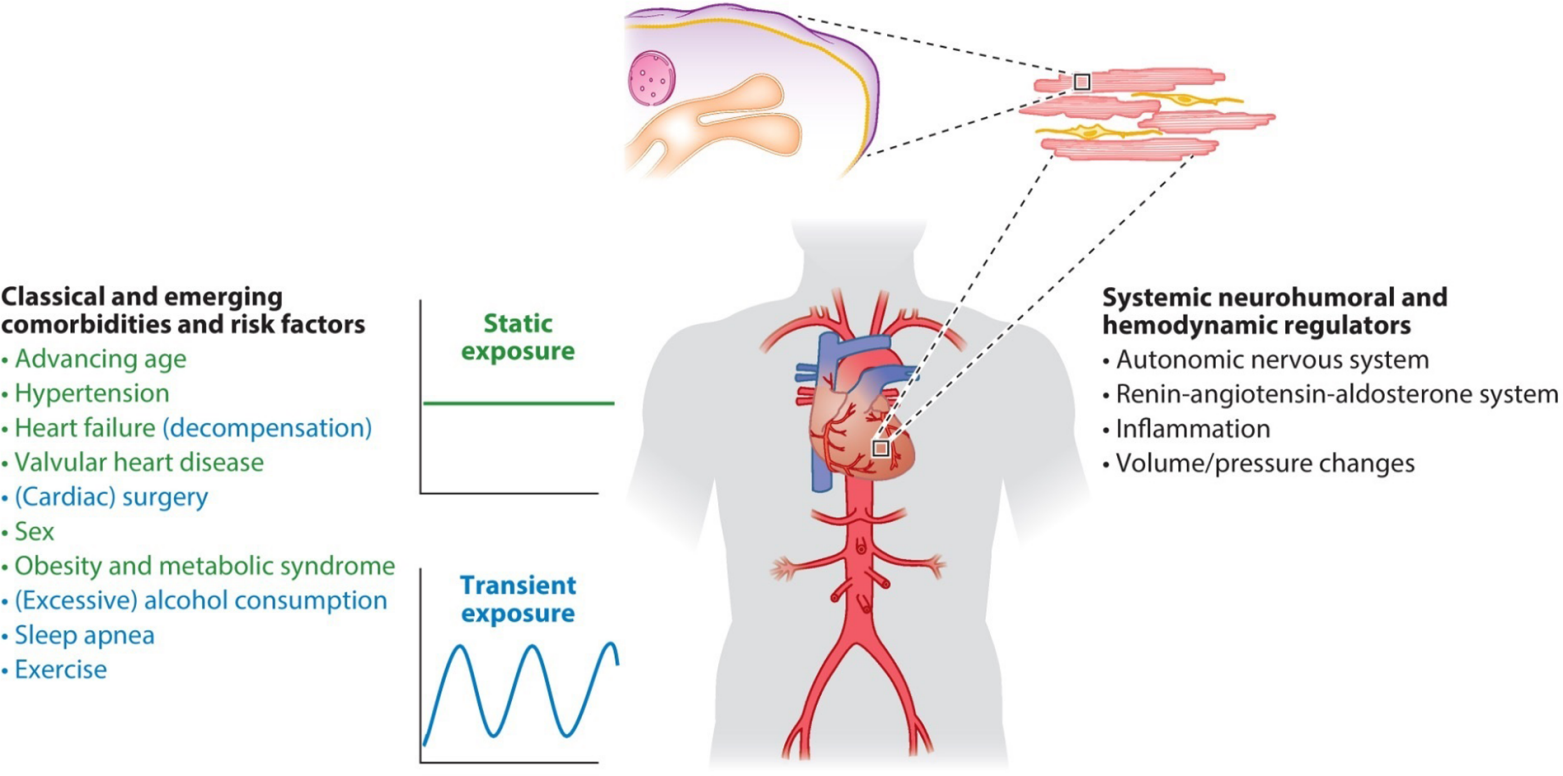
Dynamic substrate for atrial fibrillation. Reproduced from reference (235) with permission.

Hence, risk factors contribute to both a progressive AF-promoting substrate and transient changes in AF risk. Components of static risk exposure and transient risk exposure are illustrated in Figure 5 (235).

## Managing potentially reversible AF risk factors

**Figure 6:**
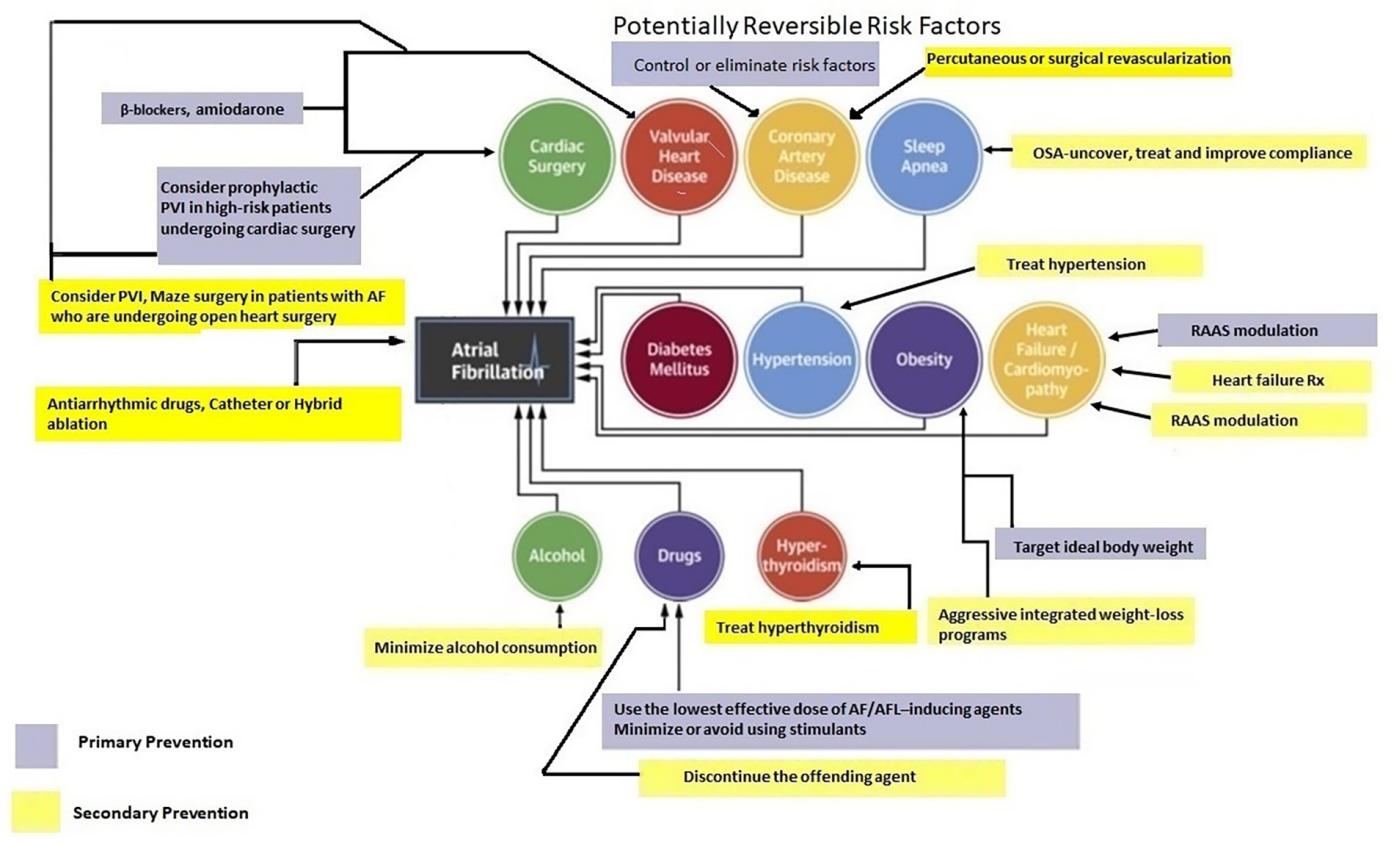
Potentially reversible risk factors. Adapted from reference (236) with permission.

Figure 6 outlines primary and secondary approaches to potentially reversible risk factors for AF and includes the roles of catheter, surgical and hybrid ablative therapies (236). A detailed discussion of all pharmacological agents that may precipitate AF is beyond the scope of this discussion. Readers are referred to reference (237) for additional information.

## Cardiac pacing in arrhythmia detection, primary and secondary AF prevention

Choosing an optimal pacing mode facilitates primary AF prevention and may play an important role in delaying or preventing progression of paroxysmal AF to persistent or permanent AF. Past studies consistently demonstrated a decreased frequency of AF with atrial-based (atrial or dual-chamber) vs. single-chamber right ventricular pacing in patients with sinus node dysfunction. Their findings suggested that time might be needed to see potential biological (remodeling) effects of right atrial pacing for AF prevention (238). Right ventricular pacing (RVP) has been associated with an increased incidence of AF (239). In a study population of dual chamber pacemaker recipients extracted from the MOde Selection Trial (MOST), ventricular desynchronization imposed by a high burden of right ventricular pacing (even when atrioventricular synchrony was preserved) increased the risk of AF in patients with sinus node dysfunction and a normal baseline QRS duration (240).

The efficacy of atrial based pacing (AAI or DDD modes) vs. single chamber ventricular pacing (VVI mode) has also been studied extensively with respect to multiple clinical outcomes in addition to new-onset AF, including heart failure hospitalization, stroke incidence, quality of life (QOL) and mortality. The most consistent clinical benefit of dual chamber pacing over single chamber ventricular pacing was reduction of incident AF (241).

AF-preventive algorithms have been designed to increase the baseline atrial pacing rate by overdrive-pacing the atrium, suppressing PACs or preventing pauses. They have not shown unequivocal clinical efficacy, and definitive conclusions about their merit have not been reached (242).

A variety of AF-termination [all are forms of atrial anti-tachycardia pacing (ATP)] algorithms have been tried with variable success. Termination options include Burst ATP, Burst+, Ramp ATP, and Reactive ATP (242).

Reactive ATP (Medtronic, Inc. Minneapolis, MN, USA), allows multiple deliveries upon detecting changes in rhythm regularity or cycle length, thus allowing additional ATP attempts during long atrial tachycardia/atrial flutter episodes or when AF organizes to either of these entities (242, 243). Reactive ATP (rATP) may prevent episodes from becoming sustained for hours, days, or weeks (243).

In 2015, the MINERVA Investigators demonstrated the efficacy of combining atrial preventive pacing, atrial rATP (DDDRP mode), and managed ventricular pacing (MVP), in preventing progression to persistent or permanent AF patients with bradycardia and prior atrial tachyarrhythmias (244).

In 2019, the risks of AT/AF events were compared between 4,016 patients with rATP-enabled vs. 4,016 control patients with rATP-disabled or unavailable in their device. The rATP group had significantly lower risks of AT/AF events lasting ≥1 day (HR 0.81), ≥7 days (HR 0.64), and ≥30 days (HR 0.56) compared to controls (all *P* < 0.0001) (243).

Older data has suggested the possibility that atrial pacing might increase AF. A 2008 study followed 309 recipients of cardiac resynchronization therapy for a mean of 18.1 ± 13.3 months. Slightly more than 2/3 of the patients (209 of 309, 67.6%) developed AF. Right atrial pacing percentage was associated with a greater risk of postimplant AF. The incidence of AF increased incrementally within RA pacing quartiles: 44.6%, 64.3%, 79.7%, and 81.6%, respectively (*P* < .001). Upon multivariate analysis, RA pacing quartile remained a significant predictor of post-CRT AF (hazard ratio: 1.92; 95% CI, 1.40–2.62, *P* < .001) (245).

Data from four pacemaker studies including 1,507 patients was analyzed and reported in 2011. During a mean follow-up of 14.3 ± 8.7 months, 77 patients developed AF. The AF incidence in the first (0%–32%), second (32%–66%), third (66%–89%), and fourth (89%–100%) quartiles of %AP was 1.3%, 5.3%, 5.8%, and 8.0%, respectively (*P* < 0.001). Multivariable analysis found that atrial pacing above the first quartile was associated with a relative risk of 2.93 (95% CI, 1.16–7.39, *P* = 0.023) (246). Algorithms to minimize atrial pacing may help prevent AF.

In 2008, De Voogt and Van Hemel discussed technical pitfalls that weakened the accuracy of atrial tachyarrhythmia diagnosis by pacemakers available at that time. These limitations included: (1) inappropriate sensing of AF because of variable and low voltage signals during AF, which might cause inappropriate detection of the onset and/or perpetuation of AF and other atrial arrhythmias; (2) far-field R wave sensing which might be prevented by adaptation of other pacemaker parameters which, in turn, could not only obscure far-field R wave sensing as well as every second beat of an atrial tachycardia; and 3) insufficient memory capacity of the pacemaker to store all atrial tachyarrhythmic episodes (247, 248).

More recently, Tayal and colleagues compared 16,383 pacemaker recipients free of AF 3 months after device implantation to 86,167 control patients. During a 2-year follow-up, pacemaker recipients had higher cumulative AF incidence (5.2% vs. 2.7%, *P* < 0.001). These investigators also compared 2202 pacemaker recipients to 2202 loop recorder recipients. During the 2-year follow-up, the AF incidence in the groups was 7.9% vs. 8.4% (loop vs. pacemaker). They concluded that pacemaker patients were at increased risk of being diagnosed with AF in comparison to a general cohort, likely due to continuous monitoring (249).

Ravi et al. compared the occurrence of new-onset AF and assessed AF disease progression between His bundle pacing (HBP) and RVP in 225 patients during long-term follow-up. There were 105 patients in the HBP group and 120 patients in the RVP group. There were 72 patients in the HBP group and 76 patients in the RVP group without a prior AF history (250).

Age was significantly lower (about 4 years) in the HBP group compared with the RVP group (*P* = 0.006). There was no significant difference between the two groups in the rest of their baseline characteristics. After adjustment for the confounder of age, the risk of new-onset AF remained lower (*P* = 0.046) in the HBP group. A significantly lower burden of new-onset AF was observed in HBP across all pacing burden subgroups (≥20%,≥40%,≥60%,≥80%) except for patients with His or RV pacing burden <20% where no significant difference was noted (250).

There were 44 patients in the RVP group and 33 patients in the HBP group who had a prior history of AF. Progression of AF was defined as an increase in AF burden by ≥10%. There was a trend toward lower risk of AF progression with HBP in the patients with His or RV pacing burden ≥40% (*P* = 0.072) which did not reach statistical significance (250).

Pastore and colleagues compared HBP with RV septal pacing and RV apical pacing in patients with and without a prior history of paroxysmal AF (mean follow-up 58.5 + 26.5 months). These investigators found that HBP resulted in a lower overall risk of progression to persistent or permanent AF (251) (*P* = 0.022). This finding was significant in 108 patients without a prior history of paroxysmal AF (*P* = 0.005), but was likely underpowered to reach statistical significance in the 38 patients with a prior history of paroxysmal AF (*P* = 0.086) (251).

Results from the Geisinger-Rush Conduction System Pacing Registry suggest that left bundle branch area pacing (LBBAP) has similar benefits to HBP. In patients with a ventricular pacing burden ≥20% LBBAP was associated with a lower risk of new-onset AF (≥30seconds,P=0.002). There was also a signifcant reduction in the new diagnosis of AF ≥6 min with LBBAP (*P* = 0.035) compared to conventional RVP (252).

Although AF frequently presents with symptoms, (as noted) it also may be asymptomatic (253–255). Paroxysmal atrial fibrillation (PAF), as opposed to permanent AF, is transient and infrequent. A small study of 8 patients with PAF suggested that asymptomatic PAF episodes occur much more frequently than symptomatic episodes (250, 256). Recent studies have concentrated on the frequency of asymptomatic short AF episodes, which have been termed atrial high-rate episodes (AHREs) (253).

AHREs are detected in pacemaker or implantable cardioverter defibrillator (ICD) recipients and often occur in the absence of AF diagnosed by the usual methods of electrocardiography or Holter monitoring (253, 257). The definition of AHREs refers to episodes lasting >6 min, predominately to reduce inclusion of electrical artefacts, and is usually confined to patients who do not have clinically detected AF (253, 258). A definition of 5 minutes has been used in some key studies, based on previously published data suggesting that a 5 minute cutoff excludes most episodes of oversensing (253, 259).

**Figure 7:**
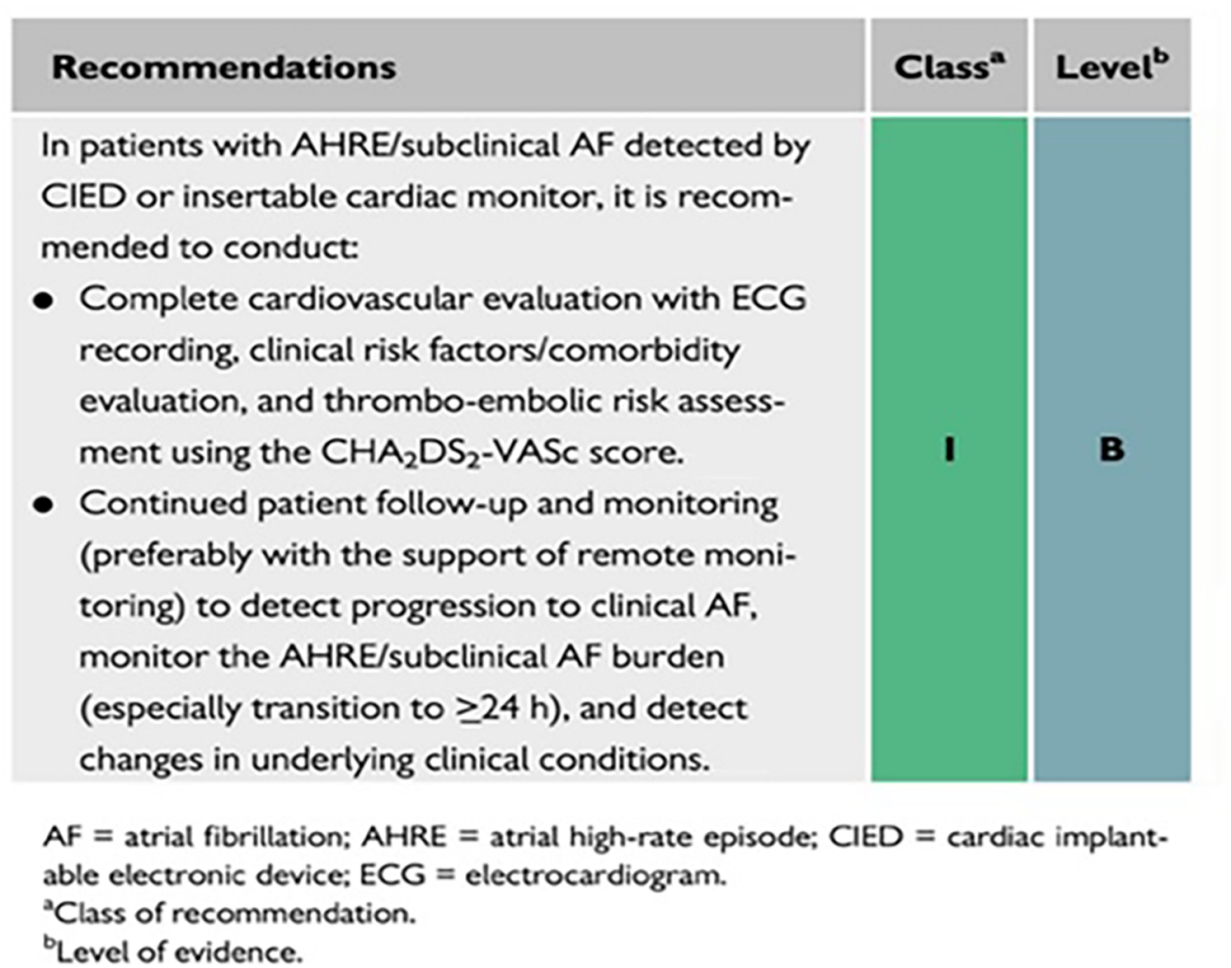
Recommendations for management of patients with AHRE. Reproduced from reference (40) with permission.

Boriani et al. analyzed pooled data from 3 prospective studies in 6,850 patients (mean age 67 ± 12, 72% male) with cardiac implanted electronic devices and no history of AF or use of anticoagulants. An AF burden of ≥5 min, was detected during a follow-up period of 2.4 ± 1.7 years in 2,244 (34%) of patients. At 36 months, the cumulative incidence of a daily AF burden of ≥5 min was 40.4% [95% confidence interval (CI): 38.9%–41.9%]. In 53% of cases the first detected AF burden ranged between 5 min and <1 h. Overall, the cumulative incidence of transition to a higher device-detected daily AF burden was 41.0% (95% CI, 38.8%–43.1%) in the first 6 months and 57.5% (95% CI, 54.8%–60.1%) at 36 months (260). Figure 7 summarizes the European Heart Rhythm Association recommendations for management of AHRE (40). Multiple studies have established a clear association between AHRE and an increased risk of stroke. These studies and their implications are summarized in Table 12 (253, 261–266).

**Table 12:** Summary of studies investigating the association between AHREs and stroke risk.

Trial	Study type and duration	Study Population	Criteria for the diagnosis of AHRE	Outcomes
MOST (261)	Subgroup analysis of RCT, 6 years	*n* = 312, median age 74, 55% female, and 60% had a history of SND	Atrial rate >220 bpm for 10 consecutive beats	Compared with controls, AHREs were associated with increased total mortality (HR 2.48; 95% CI, 1.25–4.91, *P* *=* 0.0092), death or non-fatal stroke (HR 2.79; 95% CI, 1.51–5.15, *P* *=* 0.0011), and AF (HR 5.93; 95% CI, 2.88–12.2, *P* *=* 0.0001)
TRENDS (262)	Prospective observational Study, mean follow-up 1.4 years	*n* = 2,486 with ≥1 risk factor for stroke	AT/AF burden = longest total AT/AF duration on any given day during the prior 30-day period and classified as subsets: zero, low [<5.5 h (median duration)], and high (≥5.5 h duration)	Compared with zero burden, AF burden was associated with increased TE: (HR 0.98; 95% CI, 0.34–2.82, *P* *=* 0.97) and (HR 2.20; 95% CI, 0.96–5.05, *P* *=* 0.06) for low and high, respectively
ASSERT (263)	Prospective observational Study, mean follow-up 2.5 years	*n* = 2,580, age ≥65 years, with hypertension and no history of AF	Atrial rate >190 bpm for >6 min	By 3 months, AHREs occurred in 10.1%. AHREs were associated with an increased risk of clinical AF (HR 5.56; 95% CI, 3.78–8.17; *P* *<* 0.001) and of ischemic stroke or SE (HR 2.49; 95% CI, 1.28–4.85; *P* *=* 0.007). After adjustment for predictors of stroke, AHREs remained associated with stroke/SE (HR 2.50; 95% CI, 1.28–4.89; *P* *=* 0.008)
Carelink/VA (264)	Case crossover study; analysis of data 30 days preceding a stroke	*n* = 9,850, median age 65 years, 99% male, and 98% had a defibrillator	≥5.5 h of AF on ≥1 day in the preceding 30 days	AHREs were associated with a four-fold increased risk of stroke within 30 days (OR = 4.33; 95% CI, 1.19–23.7). Risk was highest in the 5–10 days after AHRE and rapidly declined after 10 days
Belgrade (265) Atrial Fibrillation Study	Single-centre registry and mean follow-up 9.9 ± 6.1 years	*n* = 1,100, mean age 52.7 ± 12.2, 13.3% had asymptomatic AF	Asymptomatic presentation of first diagnosed AF	Ischemic stroke risk (log-rank test = 6.2, *P* *=* 0.013) was significantly worse for patients with asymptomatic AF compared with those with symptomatic AF
SOS AF (266) project	Pooled analysis of individual patient data from 5 prospective studies	*n* = 10,016, median age 70 years. Patients without permanent AF were included if they had at least 3 months of follow-up	Device-detected AF. Cutoff points of AF burden defined as: 5 min, 1, 6, 12, and 23 h	AF burden 1 h was associated with the risk of ischemic stroke (HR 2.11; 95% CI, 1.22–3.64, *P* *=* 0.008)

AF, atrial fibrillation; AHRE, atrial high-rate event; AT, atrial tachycardia; bpm, beats per minute; RCT, randomized controlled trial; SE, systemic embolism; SND, sinus node dysfunction; TE, thromboembolic event.

Adapted from reference (253) with permission.

Pacing primarily has an adjunctive role in AF therapy. In order to take advantage of the therapeutic options, physicians need intricate knowledge of device features so that precision programming can facilitate optimal patient outcomes.

## Classification of atrial fibrillation

AF is classified based on the temporal characteristics of the arrhythmia. When AF is first detected it is designated “recent-onset” (present for <48–72 h) (267, 268). AF is considered recurrent when a patient develops ≥ 2 episodes. Episodes that terminate spontaneously (by consensus within 7 days) are paroxysmal or persistent if cardioversion (electrical or pharmacological) is required to terminate AF. Successful termination of AF does not alter classification of persistent AF. Longstanding persistent AF (≥1 year) when not successfully terminated by cardioversion, or when cardioversion is not pursued, is classified as permanent (269).

## Evolving paradigms in AF management

Early in the first decade of the 21st century, several studies compared rate control and rhythm control strategies for AF. These studies suggested that a rate-control strategy would be an acceptable primary approach for patients with recurrent, persistent AF (142, 270–275) and rate control became front-line therapy in AF management.

In 2010, the RACE II Investigators reported a comparison of lenient to strict rate control in AF patients. They randomly assigned 614 permanent AF patients to undergo a lenient rate-control strategy (resting heart rate <110 beats per minute) or a strict rate-control strategy (resting heart rate <80 beats per minute and heart rate during moderate exercise <110 beats per minute). The composite primary outcome included death from a cardiovascular cause (or causes), heart failure hospitalization, stroke, systemic embolism, bleeding, and life-threatening arrhythmic events. The overall incidence as well as the components of the primary outcome were similar between the two groups. Significantly more patients in the lenient-control group met the heart-rate target or targets (*P*<0.001) and required fewer follow-up visits. Symptoms and adverse effects were similar in the two groups (276).

For most AF patients, rhythm control using available antiarrhythmic drugs was more expensive and associated with adverse drug reactions, but not more effective than the rate control strategy in preventing major adverse events. Restoration of sinus rhythm was no longer deemed imperative in asymptomatic and hemodynamically stable patients (270, 275, 276). Newer evidence has demonstrated the benefits of early intervention, either pharmacologic or ablative, and a rate control strategy should be chosen only when acceptance of the arrhythmia's permanence has been accepted by both the physician involved in the care process and the affected patient (277).

## Potential influence of the chosen rate control strategy

The ventricular rate in AF is reduced with beta blockers or nondihydropyridine calcium channel blockers (verapamil and diltiazem) and, less commonly, digoxin or amiodarone. The largest sample size and longest follow-up comparing rate control agents is a post-hoc analysis from the AFFIRM trial (278, 279). Beta-blockers were the most effective single agents. Unfortunately, drug selection was not randomized and there were significant differences between patients treated with different regimens. Beta blockers were more commonly chosen in patients with coronary disease, calcium channel blockers were more often prescribed to women and patients with pulmonary disease, digoxin was used more often in individuals with cardiomyopathy and in people of color. The impact, of these differences, on adequacy of rate control is unknown. Only 18% of patients assigned to a rate control strategy had an initial assessment of rate control adequacy at rest and with exertion. Many patients spontaneously reverted to sinus rhythm and rate control could not be assessed. Additionally, a limited number of patients in AF had their heart rate assessed with exertion (278, 279).

In a recently published *post hoc* analysis of the RACE 4 randomized trial (280, 281), the effect of rate control medication on AF progression in paroxysmal AF was analyzed. A total of 666 patients with paroxysmal AF were included in the analysis. Patients using class I or III antiarrhythmic drugs were excluded (281).

Based on evidence that non-dihydropyridine calcium channel antagonists reduce tachycardia-induced electrical remodeling in AF and that verapamil has been shown to reduce progression to persistent AF (compared to beta blockers and digoxin) in patients with vagally mediated paroxysmal AF (see below) (281–284), the authors hypothesized that this benefit might extend to the general AF population (281).

Verapamil was used in 47 patients, beta blockers in 383 and 236 patients were not using rate control drugs. The primary outcome was AF progression, defined as the need for catheter ablation, electrical or chemical cardioversion. Secondary outcomes included the individual components of the primary outcome and a composite of hospital admission for arrhythmias, heart failure, thromboembolic events, major bleeding, acute coronary syndrome, life-threatening drug effects, or cardiovascular death (280).

The verapamil group was significantly younger than the beta blocker group and had fewer men than the no rate control group. After adjustments were made for baseline characteristics, the verapamil group had a significantly lower probability of electrical or chemical cardioversion compared to the beta blocker group as well as the no rate control group. There were no significant differences in catheter ablation between groups. Likewise, there was no significant difference in the multi-component secondary outcome between the groups. The authors concluded that in patients with newly diagnosed paroxysmal AF, verapamil was associated with less progression of AF, compared to beta blockers and no rate control medication. Nevertheless, they characterized the study outcomes as hypotheses generating rather than hypothesis confirming. This cautious interpretation was based on lack of rate control treatment randomization in RACE 4, the small size of the verapamil group and the possibility of confounding factors related to differences in contraindications and adverse effects of the two drugs or after adjusting for baseline characteristics (280, 281).

## Secondary prevention: the limitations of antiarrhythmic drug therapy

Although antiarrhythmic drugs reduce AF recurrences, until recently there was little or no evidence of any benefit for other clinical outcomes compared with placebo or no treatment. A meta-analysis of 59 randomized controlled trials (20,981 patients) studied the risk and benefits of quinidine, disopyramide, propafenone, flecainide, metoprolol, amiodarone, dofetilide, dronedarone and sotalol. The outcomes analyzed included all-cause mortality, drug withdrawals due to adverse effects, proarrhythmia, stroke and AF recurrence. The authors considered study limitations, consistency of effect, imprecision, indirectness, and publication bias in order to assess the certainty of a body of evidence (270).

No antiarrhythmic drug produced a benefit on mortality. High-certainty evidence from 5 randomized controlled trials indicated that treatment with sotalol resulted in a higher all-cause mortality rate than placebo or no treatment. Quinidine was associated with a low-certainty increase in all-cause mortality compared with placebo or no treatment. Drugs with no apparent effect on mortality included metoprolol, amiodarone, dofetilide and dronedarone (270). However, two meta-analyses, focused on dronedarone and included patients with AF but also with heart failure (270, 285, 286). Both revealed a trend toward increased all-cause and cardiovascular mortality with dronedarone, compared to placebo, in this population. The more recent of these (286), suggested that dronedarone should be used cautiously as second-line medication and exclusively for the secondary prevention of paroxysmal or persistent AF, in patients without signs or symptoms of heart failure. Little or no data on mortality was available for disopyramide, flecainide or propafenone (270). In addition, it is important to note that disopyramide can cause QT prolongation and Torsade de Pointes.

Virtually all the antiarrhythmics resulted in more treatment withdrawals due to adverse effects and were associated with more proarrhythmia (the definition included severe, symptomatic bradycardia and AV block) compared with placebo or no treatment. Quinidine (at higher doses) and sotalol appeared to result in more withdrawals because of adverse events compared to controls and to other antiarrhythmic drugs. Although amiodarone compared favorably with class I agents, it had a very high relative risk (6.70) for increased withdrawal compared to placebo. These results were at 1 year of follow up. It is pivotal to remember that amiodarone's adverse effects are both dose and *time* dependent (65, 270).

Although antiarrhythmic drug therapy (including metoprolol) reduced AF recurrences by 20%–50%, AF still recurred in 43%–67% of participants treated with antiarrhythmics at one year. Amiodarone seems to be the most effective agent (65) in preventing recurrences. Nevertheless, AF recurred at one year in 43% of amiodarone treated participants (270).

Beta-blockers, sotalol, digitalis and propafenone may exacerbate episodes of vagally induced AF. In 2008, the Euro Heart Survey identified 91 patients whose AF appeared to be triggered only by vagal tone (were nocturnal or postprandial) (287). Per the 2006 AF guidelines (288), patients with vagal AF were more frequently treated with Class Ic anti-arrhythmic drugs compared with patients with adrenergic AF (*P* = 0.007). Overall, 72% of patients with vagal AF were treated with non-recommended medication, especially β-blockers (including sotalol 57%). In vagally mediated paroxysmal AF patients, non-recommended treatment was associated with deterioration to persistent or permanent AF in 19% of patients during 1-year follow-up, compared to none treated with recommended medication, however this difference did not quite reach statistical significance (*P* = 0.06) (287).

## Value of early intervention

While this evidence seems compelling, recent data suggest that early intervention with antiarrhythmic drug therapy *or* catheter ablation may be an effective approach. The EAST-AFNET 4 Trial Investigators (289) randomized 2,789 patients who had early atrial fibrillation (diagnosed ≤1 year before enrollment) to early rhythm control (1,395 assigned) vs. usual care (1,394 assigned). It should be noted that prior to this trial the main indication for rhythm control was the presence of symptoms. Patients who were randomly assigned to early rhythm-control therapy were asked to transmit a patient-operated single-lead electrocardiogram twice weekly and when symptomatic. Patients who were randomly assigned to usual care were initially treated with rate-control therapy without rhythm-control therapy. Rhythm-control therapy was added solely to mitigate uncontrolled AF–related symptoms.

The first primary outcome was a composite of cardiovascular death, stroke, or hospitalization due to deteriorating heart failure or acute coronary syndrome. The second primary outcome was the number of nights spent hospitalized per year. The primary safety outcome was a composite of death, stroke, or serious adverse events related to rhythm-control therapy. Secondary outcomes, including symptoms as well as left ventricular function, were also evaluated (289).

The first primary composite outcome was significantly reduced in the early rhythm control group. In contrast, the number of nights spent in the hospital did not differ significantly between the two groups. The percentage of patients with a primary safety outcome event did not significantly differ between the groups. Anticoagulation was maintained in 88.0% of patients assigned to early rhythm control and 90.9% of patients assigned to usual care. Likewise, symptoms and left ventricular function at 2 years did not differ significantly between the groups (289).

The authors noted that previous studies comparing rate-control and rhythm-control strategies did not show better outcomes with rhythm control compared to rate control. The authors speculated that AF ablation might have contributed to the superiority of early rhythm control in this trial (289). This seems unlikely.

Nevertheless, in the rhythm-control arm, the initial of strategy was flecainide 36%, amiodarone 20%, AF ablation 8%. By year 5 ablation accounted for only 20% of the rhythm control strategy. Results, at 5 years, from this important trial indicate that a rhythm-control strategy is superior to usual care (rate control in the majority of instances) in improving cardiovascular outcomes in patients with recently diagnosed AF and concomitant cardiovascular conditions. Results were similar in the heart failure subgroup of patients and were irrespective of symptom status. Significant reductions were noted for the primary composite endpoint, as well as for stroke and cardiovascular death. The effectiveness of early rhythm control therapy was mediated by the presence of sinus rhythm at 12 months (290).

A retrospective population-based cohort study including 22,635 Korean AF patients compared groups newly treated with rhythm control (antiarrhythmic drugs or ablation) or rate control from 2011 to 2015. When rhythm control initiated within 1 year of AF diagnosis was compared to rate control it resulted in a decreased risk of stroke. Rhythm control initiated within 6 months of AF diagnosis also reduced the risk of heart failure hospitalization. Risks of myocardial infarction and cardiovascular death did not differ between rate and rhythm control groups regardless of treatment timing (291).

## Evolving concepts in catheter ablation of AF

It is generally accepted that AF results from simultaneous reentrant wavelets. In 1998, Haïssaguerre and colleagues described the role of the pulmonary veins as an important source of ectopic beats *initiating* paroxysms of AF. Radiofrequency (RF) catheter ablation was performed at the earliest recorded site of ectopic activity within a main pulmonary vein or one of its proximal branches. The earliest local activity was marked by a “spike” (pulmonary vein potential) preceding the onset of the ectopic *P* wave. During a mean follow-up period of 8 ± 6 months, AF was completely eliminated in 62% of patients without the use of drug therapy (292).

Unfortunately, targeting focal ectopy is limited by unpredictability, inconsistent inducibility, and the risk of AF induction requiring multiple cardioversions. In addition, ablating sites within the veins presents a significant risk of pulmonary vein stenosis with a prevalence of as high as 42% (293, 294).

Embryologically the pulmonary veins originate from the posterior left atrial wall and muscular continuity between the left atrium and the tubular pulmonary veins seemed likely. Therefore, the antrum of the pulmonary veins is believed to have arrhythmogenic potential similar to the veins themselves. Recognition that pulmonary vein potentials (breakthroughs into the left atrium) could be recorded at the venous ostia allowed ostial pulmonary vein disconnection by eliminating all ostial potentials in a segmental fashion (partial perimetric ablation) (295, 296).

Segmental elimination of all ostial potentials has largely been abandoned in favor of circumferential antral pulmonary vein isolation. The circumferential antral pulmonary vein isolation procedure is easier to perform because it does not require localization of potentials and can be performed during atrial fibrillation. In addition, it reduces the likelihood of pulmonary vein stenosis. It is also more amenable to anatomic variants. Most importantly, it is more effective in preventing arrhythmic recurrence than segmental ostial isolation (71% vs. 64% for paroxysmal AF) and is a preferred ablation strategy for persistent AF (296) (see below).

Isolation of only the arrhythmogenic vein(s) has had a limited success rate. The prevalence of multiple arrhythmic pulmonary veins exceeds 70% (296, 297). Almost all pulmonary veins are capable of triggering AF. Therefore, isolation of a single arrhythmogenic vein may lead to emergence of focal ectopy from another pulmonary vein which may result in AF recurrence. Therefore, all pulmonary veins should be isolated whenever possible.

In 2000, Pappone and colleagues described an alternate technique to isolate the pulmonary veins. Because all 4 pulmonary veins were viewed as potential sources of AF, the end point was creation of circumferential lines of conduction block around each vein. Contiguous focal lesions were delivered ≥5 mm from the venous ostia. Radiofrequency energy application aimed to reduce bipolar amplitudes at each site by 80%. They used two criteria to define lesion/line continuity, low peak-to-peak bipolar potentials (amplitude during coronary sinus and right atrial pacing ≤0.1 mV) inside the circumferential lesions and local endocardial activation times during atrial pacing >30 ms between contiguous points lying in the same axial plane on the external and internal sides of lesions encircling each vein. After a follow-up period of 9 ± 3 months of follow-up, 22/26 patients (85%) had stable sinus rhythm (62% without antiarrhythmic drugs, 23% on pharmacologic therapy) (298).

Although these authors reported no outcome differences between patients with paroxysmal and permanent AF, long term success rates with circumferential left atrial antral ablation have ranged from 59%–89% for paroxysmal AF and 50%–70% for persistent AF (296).

Encircling of the pulmonary veins has been referred to as wide area circumferential ablation (298), wide antral circumferential ablation (WACA) (299), or wide area left atrial ablation (296) and may be accomplished by encircling the ipsilateral inferior and superior veins with one loop on the right and another on the left. While some studies reported that pulmonary vein isolation was not required for procedural success, complete pulmonary vein isolation improves the success rate of circumferential left atrial ablation (296, 299–301). Therefore, regardless of which energy source is used, isolation of the pulmonary veins is recommended.

Pulmonary vein isolation alone may be insufficient for treatment of patients with persistent AF. The posterior left atrial region encompassed by the LA roof, superiorly, the left and the right PVs, laterally, and the plane extending from the lower borders of the left and the right inferior PVs, form the PV component of the left atrial posterior wall, which has been implicated in genesis and maintenance of persistent AF (302). Surgical literature has suggested that isolating the “box” between the PVs in the left atrial posterior wall (LAPW) is associated with improved AF outcomes (302).

Catheter-based isolation of the left atrial posterior wall may provide additional benefit over pulmonary vein antral isolation alone in treatment of persistent AF. Posterior wall isolation (PWI) debulks the potential substrate. In addition, posterior wall isolation creates conduction block to prevent potential reentrant circuits and reduces the likelihood of small gaps in the posterior aspect of pulmonary vein isolation lesion sets which may result in AF triggers from conduction recovery. PWI may lead to more reliable ablation of ganglionated plexi (303). In addition, (as implied above) the posterior wall has embryologic links and is histologically similar to pulmonary venous tissue (304). This concept is controversial and will be discussed further under ablation technologies. There is no definite evidence that ablative techniques beyond pulmonary vein isolation improves outcomes beyond what can be achieved *via* pulmonary vein isolation.

## Catheter ablation as first-line therapy for paroxysmal AF

Past clinical guidelines have recommended a minimum of one trial of antiarrhythmic drug therapy before considering ablation therapy for AF. However, the 2014 AHA/ACC/HRS guidelines for the management of AF patients added a class IIa recommendation that, after weighing the outcomes and risks of drug and ablation therapy, catheter ablation is a reasonable initial rhythm-control strategy in symptomatic patients with, recurrent paroxysmal AF (305).

Two recent studies compared cryoablation to drug therapy for initial AF treatment (306, 307). In one multicenter study, Andrade and colleagues (306) randomly assigned (∼1:1 ratio) 303 patients with symptomatic, paroxysmal, untreated AF to undergo cryoballoon ablation or to receive antiarrhythmic drug therapy. Flecainide was the was the most frequently prescribed drug for initial rhythm control followed by sotalol, propafenone, and dronedarone. Amiodarone was employed only as a second or third drug option.

All patients had an implantable cardiac monitor (Reveal LINQ, Medtronic, Minneapolis MN) inserted within 24 h of therapy initiation. The primary end point was the first documented recurrence of any atrial tachyarrhythmia lasting ≥30 seconds between 91 and 365 days (a 90 day “blanking” period was included) after starting antiarrhythmic drug therapy or completing the catheter ablation procedure. Secondary end points included freedom from symptomatic arrhythmia, AF burden, and quality of life. Adverse events were considered serious if they resulted in death or functional disability, warranted an intervention, or resulted in a hospital stay >24 h (306).

After 1 year of follow-up, recurrence of atrial tachyarrhythmia occurred in 42.9% of cryoablation recipients and 67.8% of patients assigned to receive antiarrhythmic drugs (*P* < 0.001). Symptomatic atrial tachyarrhythmia recurred in 11.0% of the ablation cohort and 26.2% of patients assigned to receive antiarrhythmic drugs ((hazard ratio, 0.39; 95% CI, 0.22 to 0.68). The median percentage of total time in AF was 0% (interquartile range, 0 to 0.08) in patients who underwent catheter ablation and 0.13% (interquartile range, 0 to 1.60) in those assigned to the antiarrhythmic drug cohort. Quality of life scores improved more in the ablation group (higher scores indicated better health-related quality of life). Serious adverse events occurred in 5 ablation group patients (three had phrenic nerve palsy which resolved within one month) and 6 patients in the antiarrhythmic drug group (306).

The authors acknowledged that invasive procedures are associated with more up-front risk than medical therapy, the AF burden was similar in both groups and that the follow-up duration was limited to one year. Nevertheless, they concluded that cryoballoon ablation resulted in a significantly lower rate of recurrent atrial tachyarrhythmias compared to antiarrhythmic drug therapy (306).

Wazni et al. performed a similar multicenter study (307), enrolling 203 patients with paroxysmal AF who were randomly assigned (∼1:1 ratio) to receive antiarrhythmic drugs (class I or III agents) or cryoballoon pulmonary vein isolation. A twelve-lead electrocardiogram (ECG) was recorded at baseline and at 1,3,6 and 12 months. After a 90-day blanking period, patient-activated trans-telephonic monitoring was conducted weekly (and whenever symptoms occurred) during months 3 through 12. Ambulatory 24-hour monitoring was performed at 6 and 12 months.

The primary end point was treatment success at 12 months, defined as freedom from initial procedural failure; any subsequent AF surgery or left atrial ablation (including procedures performed during the blanking period); or atrial arrhythmia recurrence (atrial fibrillation, atrial flutter, or atrial tachycardia ≥30 seconds in duration documented *via* ambulatory monitoring or for ≥10 seconds on a 12-lead ECG), cardioversion, or use of class I or III antiarrhythmic drugs (ablation group only) beyond the 90-day blanking period (307).

The composite primary safety end point (evaluated solely in the ablation group) included the following serious procedure-related or cryoballoon system-related adverse events: clinically significant pericardial effusion within 30 days, atrial–esophageal fistula or symptomatic pulmonary vein stenosis within 12 months, phrenic nerve injury that had not recovered at 12 months, transient ischemic attack, stroke, myocardial infarction, major vascular complication, or major bleeding within 7 days post-procedure (102). Prespecified secondary end points included a quality-of life comparison (also evaluated only in the ablation group) between baseline and 12 months post-procedure and a health care utilization comparison between the two treatment groups (307).

Treatment success rates at 12 months were 74.6% [95% confidence interval (CI), 65.0 to 82.0] in the ablation group and 45.0% (95% CI, 34.6 to 54.7) in the drug-therapy group (*P* < 0.001). A post hoc analysis of the 78 patients in the drug-therapy group who took a therapeutic dose of an antiarrhythmic drug throughout this trial revealed treatment success in 40 patients (51%) at 1 year. Although only 1.9% of patients in the ablation group had a primary safety event at 12 months, serious adverse events occurred in 14% of patients in each study group. Quality of life improved significantly from baseline in the ablation group at 12 months. Health care utilization did not differ significantly between the groups (307).

The authors concluded that “cryoballoon ablation was superior to antiarrhythmic drug therapy for the prevention of atrial arrhythmia recurrence in patients with paroxysmal atrial fibrillation”. Nevertheless, they acknowledged several study limitations including follow-up limited to 1 year and the possibility that some patients [in the antiarrhythmic drug group] may have been undertreated, which might have increased the comparative benefit of ablation (307).

A recent meta-analysis of 997 AF patients (98% paroxysmal) from five randomized trials revealed that, compared with anti-arrhythmic drugs, catheter as first-line therapy was associated with significantly higher freedom from arrhythmia recurrence (69% vs. 48%, *P*  <  0.001). This significance was maintained in subgroup analyses of 1- and 2-year follow-up (*P*  <  0.001). Catheter ablation was associated with significantly greater improvement in QoL scores. The incidence of serious adverse events was similar between ablation and AADs group (5.6% vs. 4.9%, *P*  =  0.62) (308).

## Antiarrhythmic drugs versus ablation as first-line choices

While the data from Andrade et al. and Wazni is impressive and suggests superiority of catheter ablation as initial therapy, data from the EAST-AFNET 4 Trial Investigators (289, 290) and the very large Korean population-based cohort study (291), strongly suggest that antiarrhythmic drugs remain valuable in AF management. We believe the take home message is that a rhythm control choice should be made quickly, before AF becomes persistent and that rate control strategies should employed almost exclusively when a shared decision has been made between patient and physician that AF is permanent.

## Conclusion

In part 1 of this treatise, we have discussed the pathophysiology, epidemiology, risk factors and comorbidities associated with AF. We have commented on diet, lifestyle modifications, and pharmacological interventions for primary and secondary AF prevention/burden reduction, the benefits of antiarrhythmic drugs for secondary AF prevention/burden reduction, as well as catheter ablation for paroxysmal and persistent AF. We have outlined the benefits of early intervention for secondary AF prevention/burden reduction and reducing the risks of stroke, death, and hospitalization. In part 2, we will discuss the role of AF catheter ablation in heart failure, evolving AF catheter ablation technologies, surgical and hybrid AF ablation, as well as prevention of thromboembolic complications related to AF.

## Author contributions

HH, PS and RGT had full access to all of the data in the study and take responsibility for the integrity of the data and the accuracy of the data analysis. Study concept and design: RGT. Acquisition of data: RGT, HH, PS. Analysis and interpretation of data: RGT, HH, PS. Drafting of the manuscript: RGT. Critical revision of the manuscript for important intellectual content: HH, PS. Administrative, technical, or material support: RGT. Study supervision: RGT. All authors contributed to the article and approved the submitted version.

## Conflict of interest

RGT reports serving as an advisor to Boston Scientific/Guidant; receiving research grants from Boston Scientific/Guidant, Medtronic Inc, St Jude Medical (Abbott), Vitatron, and Wyeth-Ayerst/Wyeth Pharmaceuticals; serving as a consultant for Biosense Webster, Alta Thera Pharmaceuticals, and Newron Pharmaceuticals P.s.A.; and receiving speakers fees or honoraria from Boston Scientific/Guidant CRM, Medtronic Inc, Alta Thera Pharmaceuticals, Daichii Sankyo and St Jude Medical (Abbott). The remaining authors declare that the research was conducted in the absence of any commercial or financial relationships that could be construed as a potential conflict of interest.
